# Comparing apples and oranges in youth depression treatments? A quantitative critique of the evidence base and guidelines

**DOI:** 10.1136/bmjment-2024-301162

**Published:** 2025-01-19

**Authors:** Argyris Stringaris, Charlotte Burman, Raphaelle Delpech, Rudolph Uher, Dayna Bhudia, Despoina Miliou, Ioannis-Marios Rokas, Marinos Kyriakopoulos, Lucy Foulkes, Carmen Moreno, Samuele Cortese, Glyn Lewis, Georgina Krebs

**Affiliations:** 1 University College London, London, UK; 2 National and Kapodistrian University of Athens, Athens, Attica, Greece; 3 Dalhousie University, Halifax, Nova Scotia, Canada; 4 King's College London, London, UK; 5 Child and Adolescent Mental Health Services, South London and Maudsley NHS Foundation Trust, London, UK; 6 Department of Experimental Psychology, University of Oxford, Oxford, UK; 7 Institute of Psychiatry and Mental Health, Hospital General Universitario Gregorio Marañón, IiSGM, CIBERSAM, ISCIII, Hospital General Universitario Gregorio Marañón, Madrid, Spain; 8 Psychiatry, Universidad Complutense de Madrid Facultad de Medicina, Madrid, Spain; 9 University of Southampton, Southampton, UK

**Keywords:** Child & adolescent psychiatry, Depression, Data Interpretation, Statistical

## Abstract

**Objectives:**

Should a young person receive psychotherapy or medication for their depression and on what evidence do we base this decision? In this paper, we test the factors across modalities that may influence comparability between medication and psychotherapy trials.

**Methods:**

We included 92 randomised controlled trials (RCTs) of psychotherapy and medication for child and adolescent depression (mean age 4–18 years). Using meta-analyses, we compared (a) participant characteristics and (b) trial characteristics in medication and psychotherapy trials. Lastly, we examined whether psychotherapy controls are well-matched to active conditions.

**Results:**

Participants in medication RCTs had higher depression severity and were more frequently male compared with psychotherapy RCTs. There was a dramatic difference in the within-subject improvement due to placebo (SMD=−1.9 (95% CI: −2.1 to −1.7)) vs. psychotherapy controls (SMD=−0.6 (95% CI: −0.9 to −0.3)). Within psychotherapy RCTs, control conditions were less intensive on average than active conditions.

**Conclusions:**

Medication and psychotherapy RCTs differ on fundamental participant and methodological characteristics, thereby raising questions about their comparability. Psychotherapy controls often involve little therapist contact and are easy-to-beat comparators. These findings cast doubt on the confidence with which psychotherapy is recommended for youth depression and highlight the pressing need to improve the evidence base.

**WHAT IS ALREADY KNOWN ON THIS TOPIC**

- Psychotherapy is recommended before medication for most cases of depression in children and adolescents, a recommendation that is based on indirect comparisons of outcomes from randomised controlled trials (RCTs) within each treatment modality.

**WHAT THIS STUDY ADDS**

- We examine the validity of these inferences by scrutinising the comparability of psychotherapy and medication RCTs.
- We find significant differences in sample characteristics (namely depression severity and sex composition) and trial design features, such that the within-group effect sizes of medication controls (ie, pill placebo) are much larger than those for psychotherapy controls and that medication RCTs feature significantly more trial sites.
- We also examine the quality of controls used in psychotherapy RCTs and find that they are poorly matched to active intervention arms in ways such as human contact hours and hence represent poor and easy-to-beat comparators.

**HOW THIS STUDY MIGHT AFFECT RESEARCH, PRACTICE OR POLICY**

- Our findings underscore the need for higher quality evidence on which to base treatment guidelines and clinical decision-making.

## Background

 Should a child or an adolescent receive psychotherapy or medication for their depression, and what information should be used to guide decision-making?

For adolescent depression, there are limited head-to-head trials of medication and psychotherapy, and hence recommendations are derived from indirect comparisons of treatment efficacy. The National Institute of Health and Care Excellence (NICE) guidelines for adolescent depression recommend psychotherapy over medication in most cases.^1^ This is in keeping with two sources of evidence relating to child and adolescent depression: meta-analyses of medication randomised controlled trials (RCTs) that cast doubt on the efficacy of antidepressants, with the exception of fluoxetine,^2^ and meta-analyses of psychotherapy RCTs that conclude psychotherapy to be efficacious.^3^ However, a recent network meta-analysis (NMA),^4^ an established method of comparing treatments using both direct and indirect (ie, treatment A with treatment C, via studies that directly compare A with B and B with C) evidence, concluded that only fluoxetine alone and fluoxetine administered together with CBT were significantly more effective than medication controls (ie, pill placebo) or psychotherapy controls. A large head-to-head RCT comparing modalities found that fluoxetine, alone and in combination with CBT, was superior to pill placebo, although CBT alone was not.^5^ Also, the addition of psychotherapy to standard care did not improve outcomes.^6^ Given this confusing evidence base, how should we make treatment decisions?

In this paper, we examine whether the existing evidence for adolescent depression treatments can offer valid answers to this question. We provide a conceptual framework and test a series of hypotheses using data from existing trials. Two points are crucial to indirect comparisons of treatment modalities. First, whether the participants in trials are comparable across modalities or differ in potential effect modifiers. Second, whether key conditions of the trial, such as the effects of control conditions or the number of sites involved, are comparable.

Starting with the first point, comparison between different trials assumes that they sample from populations that are comparable in terms of characteristics that could be effect modifiers. If not, the validity of any comparisons, including those conducted through NMA (which rests on the principle of transitivity, ie the requirement that the different sets of randomised trials are similar on average),^7^ is questionable.

The assumption that medication and psychotherapy trials sample from comparable population may not be valid as patients and parents often have treatment preferences,^8^,^10^ meaning that there is likely to be a self-selection bias in who participates in psychotherapy and medication trials. Moreover, treatment preferences correlate with clinically relevant participant characteristics, including severity and sex. Some of these characteristics, such as severity, may moderate treatment response^11 12^ and may confound comparisons.

Regarding the second point, differences in trial design may impact outcomes in a differential way between medication and psychotherapy trials.^13^ Most obviously, participants in psychotherapy trials are generally unblinded to treatment allocation, with the exception perhaps of trials that compare two equally plausible treatment arms.^14^ This creates differential expectations, which may favour the psychotherapy active condition, as participants are content to be receiving the ‘cutting edge’ treatment, while those in the control are dissatisfied for having missed out (ie, ‘disappointment bias’).^15^ By contrast, in new antidepressant trials, patients (and raters) were largely unable to judge treatment allocation,^16^ suggesting that expectancy effects are well-matched across conditions. Since expectancy is substantially associated with treatment outcomes,^17^ if expectancy differs between medication and psychotherapy trials, comparisons between them, including in NMA, become questionable.

Another difference in design is the number of trial sites. The number of sites in medication trials is positively related to the magnitude of the placebo response.^18^,^20^ This phenomenon may be due to the lower quality of assessments in multi-site trials, with higher rates of classification errors and therefore higher apparent spontaneous remission or regression to the mean.

An inter-related issue concerns the effect of control conditions. Often psychotherapy and medication are compared on the basis of their respective effect sizes (ie, differences between the active and the control conditions for each modality). For these to be comparable, medication and psychotherapy controls ought to be equal in their effects. Otherwise, misleading conclusions could be drawn; for example, two effect sizes of 40% would be considered equal, even if one arose from a difference of 100% vs 60% and another from a difference of 40% vs 0% (i.e., from different points of reference).

Additionally, control conditions in RCTs should generate counterfactual conditions to the intervention: what would have been the outcome had an individual not received the intervention, with all else being equal?^21^ Pill placebo, where the appearance of the drug is faithfully emulated, is an effort for all else to be equal. In psychotherapy trials, control conditions may not be so well-matched to the intervention (eg, in the number of contact hours).

## Objective

We examine RCTs of psychotherapy and medication for child and adolescent depression (mean age 4–18 years). We posit there are substantial differences between psychotherapy and medication RCTs, making their comparison problematic and examine the following: first, we conduct meta-analyses to compare sample characteristics of medication and psychotherapy trials including: (a) baseline depression severity, (b) percentage of females, and (c) mean age. Second, we examine trial characteristics including the efficacy of the control arms, using random-effects meta-regression, and the number of trial sites. Third, we scrutinise the extent to which psychotherapy controls matched the active intervention in ways such as the number and frequency of sessions, and hence whether they represent fair pairings from which to draw valid efficacy inferences.

## Study selection and analysis

The protocol was registered on the Open Science Framework (deviations in online supplemental table S1).^22^ A detailed description of our methods can be found in the online supplemental file.

### Included studies

We included RCTs identified in a recent meta-analysis of psychotherapy vs. control,^3^ an NMA examining the efficacy of antidepressants^2^ and an NMA comparing both treatment types^4^ for depression in children and adolescents. For the psychotherapy trials, we used open data from the previous meta-analysis.^23^ For medication trials, we were unable to access the full dataset used in the NMA and hence extracted data from the included studies ourselves.

For medication trials, we also conducted a systematic search for studies published after the final search date of the review of Cipriani *et al*^2^ up to the final search date of the review of Cuijpers *et al*^3^ to ensure we analysed an equivalently up-to-date database of medication trials. Two authors screened 450 titles and abstracts, and 38 full text records. Seven studies met inclusion criteria and one author completed data extraction for these papers.

### Statistical analysis

#### Sample characteristics

We conducted random-effects meta-analyses and tested subgroup differences (psychotherapy vs. medication trials) in severity of depressive symptoms, sex and age. Meta-analyses were implemented using R’s *meta* package.

#### Trial design

##### Measures of effect

As the measure of the effect of each individual study, we used the within-group standardised mean difference (SMD) for the primary depression scale used (selected using the hierarchy in the online supplemental file).

Where individual studies did not report all data required to calculate the SMD, we imputed missing data according to the methods summarised in the Cochrane Handbook.^24^

For meta-analysis, it is necessary to estimate an SE of the SMD. This requires a correlation between the pre-measures and the post-measures, a statistic typically not reported. To ensure that our results are not biased by misestimation,^25^ we simulated n=1000 datasets for different values (0.45–0.9) of this correlation and used these datasets in subsequent analyses.

##### Multilevel model metaregression

We estimated the pooled SMD for each arm by using multilevel models implemented in R’s *metafor* package.

We present the SMDs of each of the four treatment arms (medication control, medication active, psychotherapy control, psychotherapy active) under investigation. The SMDs are the means across the 1000 simulated datasets.

##### Number of sites

We also conducted a t-test to compare the mean number of trial sites between psychotherapy and medication trials.

##### Sensitivity analyses

We conducted sensitivity analyses where we excluded studies that used waitlist as their control and recruited participants with subclinical levels of depression. Next, we included only trials that used the Children’s Depression Rating Scale, Revised (CDRS-R) or the Hamilton Depression Rating Scale (HAM-D) as outcome instruments. Next, we restricted the analysis to studies with variance below 0.02. Further, we tested whether simulated values for the SE had a substantial influence on the estimation of the differences between the medication and psychotherapy control conditions. We plotted the z-value of the difference between the two coefficients against the number of simulations. We make an inference on the stability of the difference by counting the proportion of times that the z-value is above the critical value of z=1.645 corresponding to an alpha=0.05.

Finally, we examined whether differential regression to the mean may account for differences in effect for psychotherapy and medication trials.

### Comparing the control and active arms of psychotherapy trials

We ran t-tests to compare the active and control arms of psychotherapy trials on key variables of interest regarding the intensity of the interventions: the number, duration and intensity of sessions, and the total cumulative hours and duration of the intervention.

## Findings

### Included studies

Data for included studies are summarised in online supplemental table S2 and available on the project repository.^26^

**Figure 1:**
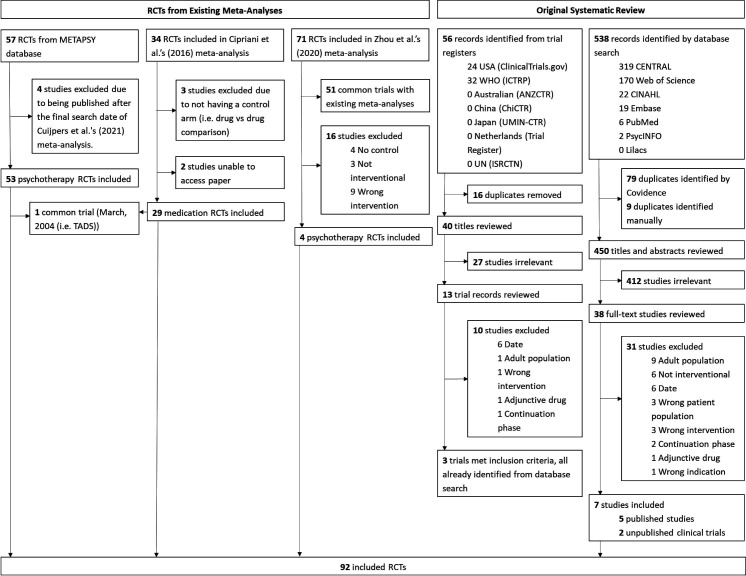
Preferred Reporting Items for Systematic Reviews and Meta-Analyses chart summarising sources of the included studies. RCTs, randomised controlled trials; TADS, Treatment for Adolescents With Depression Study.

In total, there were 92 RCTs, which included 48 active arms and 36 control arms of medication trials and 67 active arms and 62 control arms from psychotherapy RCTs (see figure 1 for a summary of sources). Note that the number of active and control arms does not match because some studies feature more than one control or active arm.

Placebo pill was the control condition for all medication trials. In psychotherapy trials, the control arm included 14 waitlists, 28 treatment-as-usual (TAU) and 20 other control conditions.

### Sample characteristics at baseline in medication and psychotherapy trials

Table 1 summarises the results from each of the meta-analyses examining sample characteristics at baseline. The summary statistics are provided for each subgroup, and the p-value is derived from the test for subgroup differences.

**Table 1:** Sample characteristics at baseline across medication and psychotherapy studies: results for overall sample and sensitivity analyses

Subgroup	K	Mean	SE	Lower CI	Upper CI	T^2^	P value
Baseline Severity of Depressive Symptoms*							
Overall							0.033
Psychotherapy	49	0.37	0.02	0.33	0.41	0.02	
Medication	31	0.42	0.01	0.39	0.44	0	
Excluding subclinical							0.281
Psychotherapy	41	0.39	0.02	0.35	0.43	0.02	
Medication	31	0.42	0.01	0.39	0.44	0	
Excluding waitlist							0.075
Psychotherapy	41	0.37	0.02	0.33	0.42	0.02	
Medication	31	0.42	0.01	0.39	0.44	0	
Percent female							
Overall							0.020
Psychotherapy	49	61.36	2.31	56.72	66.00	260.97	
Medication	28	53.72	2.33	48.94	58.51	152.15	
Excluding subclinical							0.035
Psychotherapy	42	61.72	2.63	56.41	67.02	289.77	
Medication	28	53.72	2.33	48.94	58.51	152.15	
Excluding waitlist							0.044
Psychotherapy	41	61.38	2.60	56.12	66.63	277.58	
Medication	28	53.72	2.33	48.94	58.51	152.15	
Age							
Overall							0.220
Psychotherapy	53	14.3	0.33	13.64	14.96	5.7	
Medication	28	13.69	0.37	12.95	14.44	3.7	
Excluding subclinical							0.249
Psychotherapy	44	14.29	0.37	13.55	15.04	5.98	
Medication	28	13.69	0.37	12.95	14.44	3.7	
Excluding waitlist							0.249
Psychotherapy	45	14.29	0.36	13.56	15.01	5.82	
Medication	28	13.69	0.37	12.95	14.44	3.7	

* These are baseline depression scores transformed to reflect percentage of a scale range (see online supplemental file for detailed description). To take an example, the CDRS gives a possible total score from 17 to 113 (i.e., range of 96). Mean severity was 0.36 for psychotherapy studies and 0.42 for medication studies, which would translate to 51.56 (17 + 0.36 x 96) and 57.32 (17 + 0.42 x 96), respectively, as equivalent scores on the CDRS.

CDRSChildren’s Depression Rating ScaleKnumber of studiesT2estimate of between-study heterogeneity

#### Baseline severity

On average, depression severity at baseline was significantly higher in medication trials compared with psychotherapy trials (see table 1). When excluding RCTs that used a waitlist as their control, baseline severity remained significantly higher in medication trials compared with psychotherapy trials. This difference did not reach statistical significance when excluding studies that recruited samples with subclinical depression.

To ensure that this was not an artefact of variable transformation, we also compared means at baseline in the two instruments, CDRS and HAM-D, on which there was a sufficient number of studies to meta-analyse. As can be seen in online supplemental table S3 and table S4, the number of studies is much smaller, but the pattern of differences is the same for the HAM-D and the CDRS, though it does not reach statistical significance for the latter.

#### Sex

For this analysis, we excluded the two psychotherapy trials that included entirely female samples (Moeini, 2019; Shomaker, 2016; see online supplemental table S5 for results including all studies). As can be seen in table 1, psychotherapy trials featured a significantly higher percentage of females when compared with medication trials. On average, samples were 61.36% (SE=2.31) female across psychotherapy trials and 53.72% (SE=2.33) female across medication trials. Excluding subclinical and waitlist control studies yielded similar results.

#### Age

As can be seen in table 1, the mean age was 14.3 (SE=0.33) across psychotherapy trials and 13.7 (SE=0.37) across medication trials, with no significant between-group differences. There were no significant differences in mean age between modalities on further sensitivity analyses.

### Trial design

#### Standardised mean differences of control conditions in psychotherapy and medication studies

**Figure 2:**
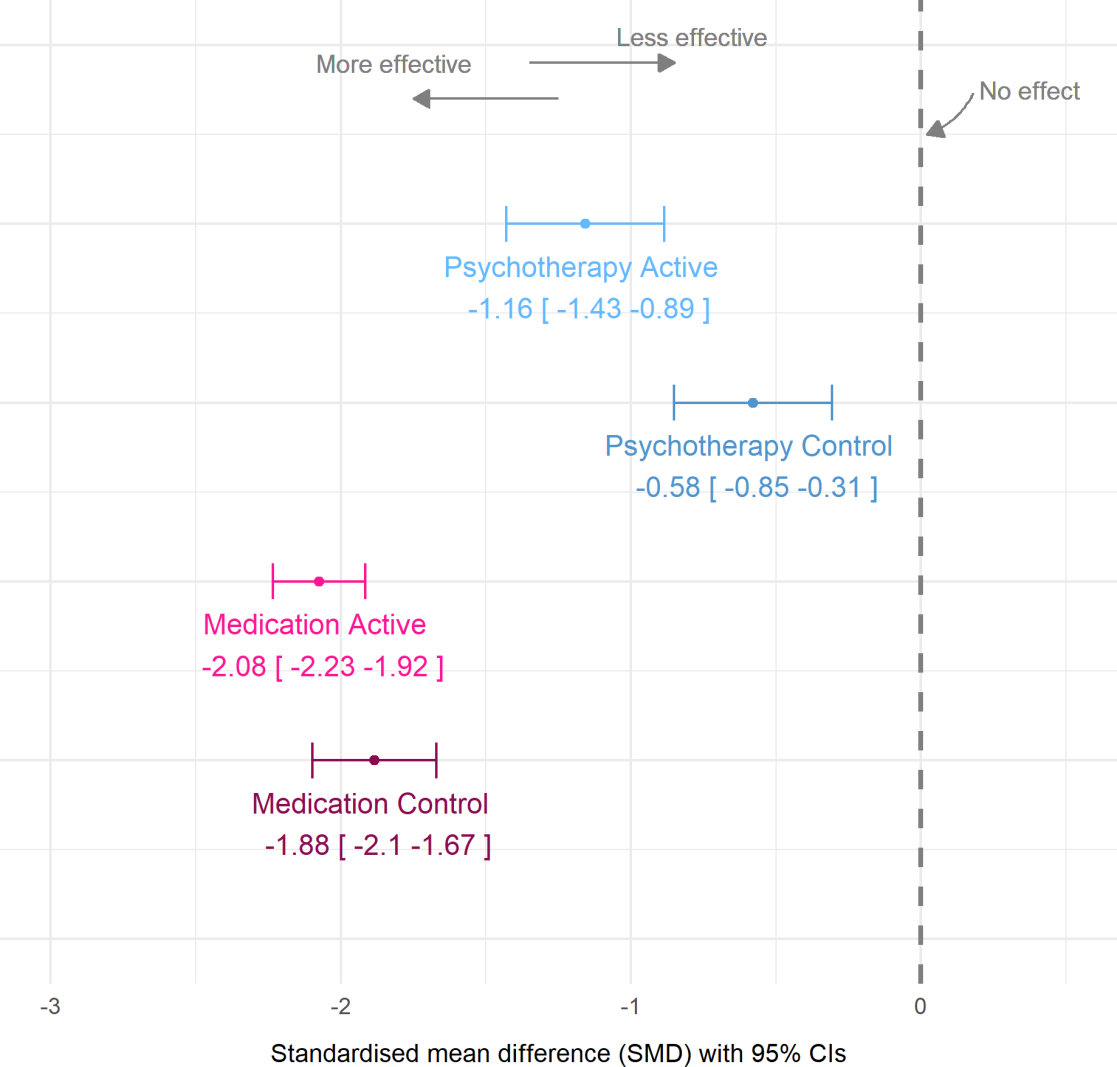
Meta-analytic estimates of within-group changes in active and control arms of medication and psychotherapy trials.

**Figure 3:**
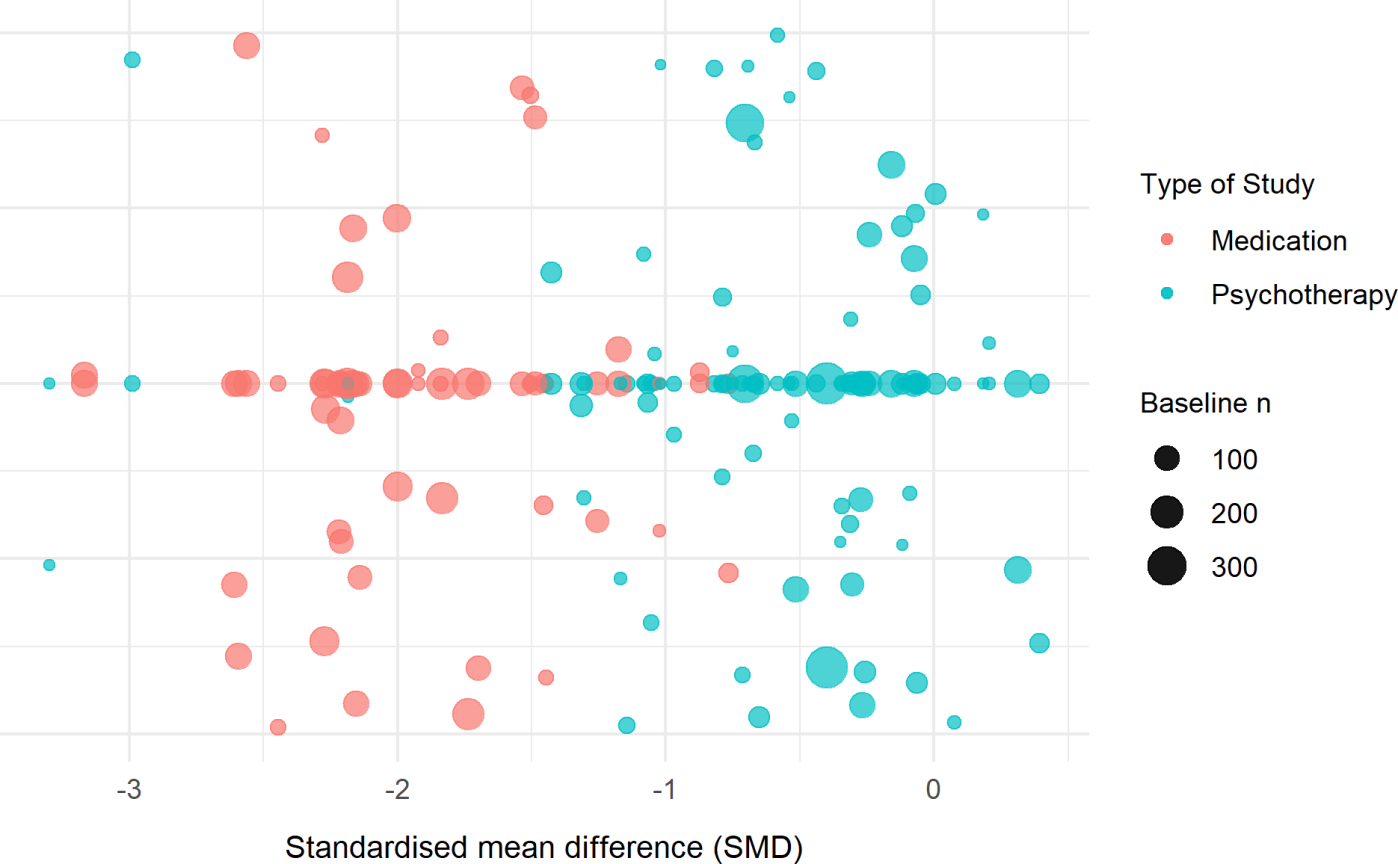
Weighted scatterplot depicting the pre–post standardised mean differences (SMD) of control arms in medication and psychotherapy trials.

We applied metaregression to obtain the SMDs and CIs of each of the four study arms (see online supplemental table S6 for full results). As seen in figure 2, there were substantial differences between the four arms of the meta-analysis with striking differences between the medication and the psychotherapy control arms (see figure 3 for weighted scatterplot; see online supplemental figure S1,S2 for post–pre differences in mean scores and variances). In particular, the pill placebo had an SMD=−1.9 (95% CI: −2.1 to −1.7), whereas psychotherapy controls had an SMD=−0.6 (95% CI: −0.9 to −0.3).

#### Sensitivity analyses

We conducted a series of sensitivity analyses. Excluding waitlist control studies (see online supplemental figure S3) and subclinical studies (see online supplemental figure S4) yielded a pattern of results very similar to the overall analyses. Next, we examined the data including only those studies that used the CDRS (see online supplemental figure S5) or the HAMD (see online supplemental figure S6). Medication control and psychotherapy control conditions remained significantly different, though the small number of studies resulted in less precise estimates of the SMDs. Restricting the analysis to studies reporting post SDs, or studies with variance below 0.02, yielded a similar pattern of results but with increased precision in SMD estimates for the latter (see online supplemental figures S7,S8). Finally, we showed that different values for the pre–post measure correlation had minimal effect on the estimated outcomes (see online supplemental figure S9).

#### Regression to the mean

We addressed potential regression to the mean by including the baseline score for each depression scale in the linear regression model as per equation 3 in Barnett *et al*^27^ (see online supplemental table S7,S8). The difference between the medication control and psychotherapy control arms remained significant.

#### Number of trial sites

The average number of trial sites was significantly higher in medication trials (M=35.96, SD=25.16) compared with psychotherapy studies (M=3.04, SD=3.13) (*t*(27.51) = 6.89, p≤0.001). Of those studies with data available, 26 of 28 (93%) medication trials were multisite compared with 24 of 45 (54%) psychotherapy studies.

### Comparing the nature and intensity of control conditions in psychotherapy trials

Active conditions featured significantly more sessions compared with control conditions (see table 2). Sessions in active conditions were longer and more frequent, resulting in significantly more intervention hours overall. Notably, many control conditions were very poorly described and their intensity could not be quantified, resulting in missing data. We performed a sensitivity analysis where we excluded trials using waitlist controls; with the exception of the number of sessions, differences between active and control arms were no longer statistically significant though remained substantial (see online supplemental table S9).

**Table 2:** Comparing the intensity of active and control arms in psychotherapy studies

Group	K	Mean	SD	Cohen’s d	Upper CI	Lower CI	t	df	P value
Number of sessions									
Active	68	12.94	11.02	0.76	0.36	1.17	4.4	106.38	<0.001
Control	41	5.71	6.10						
Intensity (sessions per week)									
Active	62	1.28	0.71	1.02	0.58	1.46	4.98	79.37	<0.001
Control	37	0.58	0.67						
Session length (min)									
Active	57	65.52	31.63	1.10	0.65	1.56	5.07	68.93	<0.001
Control	36	29.12	35.01						
Total intervention hours									
Active	59	13.80	9.88	0.95	0.51	1.39	4.8	89.86	<0.001
Control	37	5.17	7.63						

Note: Statistical significance evaluated using Bonferroni-corrected criterion (α=0.05/4=0.0125).

df, degrees of freedomK, number of studies

## Conclusions and clinical implications

We examined whether psychotherapy and medication can be meaningfully compared on the basis of the existing evidence by looking at factors that influence comparability. First, whether the participants of trials in one modality are comparable with those in another modality. Second, whether the conditions of the trial, such as the effects of control conditions or the number of sites involved, are comparable.

Starting with the first question, we found that participants in medication trials are comparable in age but are more likely to be male and have more severe depression compared with those in psychotherapy. This indicates that different people enter medication and psychotherapy trials; as these could be effect modifiers, they may violate basic assumptions of comparability.

Severity is particularly important as it may moderate treatment response, with some evidence suggesting that those with higher baseline scores respond more to antidepressants^28^ or that their response to pill placebo is lower.^18^ Other studies argue against severity as a treatment moderator^29 30^; however these are within people who have chosen to be in the particular trial and modality. Moreover, severity may represent different subtypes in terms of the course of depression and real-life outcomes.^31 32^ However, our study cannot demonstrate effect modification, and it cannot be inferred that differences in participant characteristics explain observed differences in effect.

We then asked whether trial design conditions are comparable between modalities. Medication trials were more likely to be multisite than their psychotherapy counterparts: 93% of medication RCTs were multisite compared with 54% of psychotherapy RCTs. Multisite trials are associated with higher pill placebo response^18^ and are less common in publicly funded trials which show lower pill placebo efficacy.^19 20^ This aligns with medication trials being more frequently funded by pharmaceutical companies (68% in Zhou *et al*^4^), which can introduce bias in the RCT. However, in single-site trials, principal investigators are often intellectually invested in the treatment (in psychotherapy these are often treatments developed by the PI); this is in contrast to the incentive structure in multisite trials where the number of recruited participants is the primary unit of reimbursement. Concerns about allegiance bias in psychotherapy trials have been raised previously.^33^ Psychotherapy trials also tend to receive higher bias ratings compared with medication trials (e.g., 78% against 20% in Zhou *et al*^4^), further complicating comparisons.

Second, psychotherapy controls have moderate effect sizes (−0.6) whereas medication controls have very large effect sizes (−1.9). Our analysis could be critiqued for comparing within-arm symptom change per trial. This applies if we were to draw inferences about each arm’s efficacy—where preserving randomisation to balance confounders is critical. Importantly, we do not claim that these differences are genuinely due to efficacy differences; they may well be because people who attend psychotherapy and medication trials are different and respond differently. In either case, the disparity in the response to control conditions is reason for concern about our ability to draw inferences from comparisons of modalities. This is problematic as clinicians and policy-makers often resort to between-group effect sizes to summarise findings.

Our findings are largely in keeping with those of the NMA,^4^ which is designed to preserve the randomisation structure. In Zhou *et al*, the estimates for psychotherapy controls, TAU and waitlist conditions favoured placebo (though CIs were broad because these were indirectly estimated), as did estimates for psychodynamic and behavioural therapy. CBT did not differentiate from placebo, a result that is likely heavily weighted by the results of their direct comparison in the Treatment for Adolescents With Depression Study trial.^5^ It is possible that any intervention that establishes an alliance between participants and providers is equally beneficial,^34 35^ raising questions about whether specific psychological interventions with highly trained therapists are necessary. This should be considered as a null hypothesis against which to test alternatives. We note that the NMA by Zhou *et al*, an admirable effort to synthesise the literature, reports on issues that may affect transitivity with tests of incoherence showing significant differences.

We next examined whether psychotherapy controls are reasonable counterfactuals to receiving treatment. An obvious disadvantage of psychotherapy trials is that they are typically unblinded and may be inherently impossible to blind. Yet, psychotherapy trials are unlikely to fulfil other basic conditions of the ‘all else is equal’ assumption. In order to test that a psychological treatment is effective per se (e.g., because of the specific techniques) rather than because of generic effects (eg, pleasant human contact), aspects such as therapist contact time should be matched. Many (23%) psychotherapy RCTs used waitlist controls, which by definition do not match for hours of therapist contact and are often associated with disappointment bias. TAU and other psychotherapy control conditions varied drastically; 9 RCTs used controls that exactly matched the active arm in total number of contact hours, though several studies used bibliotherapy or online-only control conditions that did not involve any direct therapist contact. Importantly, controls were often poorly described, resulting in difficulties in evaluating their adequacy as counterfactual conditions. Overall, there is poor matching of control to active treatment conditions in psychotherapy RCTs, with the latter typically featuring considerably more contact hours, which may artificially inflate estimates of efficacy.

Given this, the empirical basis for comparing psychotherapy and medication for adolescent depression is weak, and hence, it is difficult to generate guidelines and recommend one treatment over another. Alternative reasons for recommending psychotherapy over medication in guidelines (eg, the presumed better side effect profile) should be clearly stated and supported by evidence. Indeed, our findings have several implications for stakeholders.

First, the grounds for comparison between medication and psychotherapy should be seen as shaky rather than offering confidence, and there is an urgent need to revisit guidelines and public information in light of the limitations.

Second, the over-reliance on easy-to-beat control conditions in psychotherapy trials should prompt consideration of how to create fair comparators. Investment should be directed into providing rigorous evidence that establishes depression psychotherapies as more efficacious than fair controls. There are examples of RCTs where such rigour has been applied in matching active and control arms on variables such as therapist time and provision of homework.^36^,^38^ Moreover, there is a place for comparing interventions to TAU since these represent real-world comparators. However, issues of disappointment bias should be addressed to avoid inflating treatment estimates.

Third, our findings make clear the inherent difficulties of comparing psychotherapy with medication trials.^13^ The first obstacle is the comparability of the populations taking part. Head-to-head comparisons of psychotherapy with medication are more favourable in this regard, yet even so, these trials might sample the population of those who are indifferent to which treatment they receive.^29^ Difficulties with blinding the psychotherapy control would also have to be overcome to draw valid inferences.

It is not surprising that in the scientific discovery process, there are complexities leading to studies with different designs and aims, and therefore to an apples and oranges situation. This does not invalidate the process as such, nor the individual studies, but does raise questions about whether such studies can be summed up and be deemed comparable. This paper is a critique of the latter point.

In summary, our results question the state of knowledge about the efficacy of psychotherapies and the extent to which giving them primacy in the treatment of depression is justified and beneficial for young people. Guidelines should not result from metanalyses on their own. Value-based judgements and conventions are key to clinical and public health practice and may put into perspective quantitative findings. Yet, there should be transparency in the decision-making. Readers of these guidelines need to be informed about the state of knowledge. In this, quantitative evidence is necessary, though insufficient by itself.

## supplementary material

10.1136/bmjment-2024-301162 online supplemental file 1

## Data Availability

Data are available in a public, open access repository. Data are available upon reasonable request.
