# Supplementary material for: Comparing apples and oranges in youth depression treatments? A quantitative critique of the evidence base and guidelines
Source: BMJ Ment Health. 2025 Jan 19;28(1):e301162. doi: 10.1136/bmjment-2024-301162 (PMC11752052; doi:10.1136/bmjment-2024-301162)
Supplement: online supplemental file 1 [file bmjment-28-1-s001.docx]

# SUPPLEMENTAL MATERIALS

# DEVIATIONS FROM PRE-REGISTRATION

The study was pre-registered on the Open Science Framework (OSF) on October 17^th^ 2023 (accessible at <https://osf.io/jxs5d>). Deviations from the original registration are detailed in *Table S1* below.

*Table S1: Deviations from pre-registered study protocol*

| **Planned approach** | **Deviation** |
| --- | --- |
| **Included studies.** Studies will be pulled from: two existing meta-analyses (Cuijpers et al., (3); Cipriani et al., (2)) and a systematic search for pharmacotherapy trials published up to the release of Cuijpers et al. | Four psychotherapy trials were included from the Zhou et al. network meta-analysis (4) in response to a reviewer’s comment. |
| **Response & deterioration rates.** The mean proportion of responders (50% reduction in symptom severity from baseline) by group (active and control) will be compared across psychotherapy and medication trials. A similar method will be used to calculate deterioration rates. | We compared within-group standardised mean differences (SMDs) instead of response rates due to the challenges in finding a uniform definition of response (and deterioration) rate. |
| **Discrete adverse effects.** We will examine differences in reported adverse effects across treatment modalities. | This aim was not included in the final manuscript due to the lack of trials reporting adverse effects. |
| **Data exclusion.** Studies will be excluded if they do not provide effect sizes, response and deterioration rates, or the data required to estimate these. | If SMDs could not be calculated from the available data in a trial, we imputed values according to the procedure in the Cochrane Handbook (20). |
| **Risk of bias.** We will use the Cochrane Risk of Bias (RoB) tool to carry out our independent assessments of the methodological quality of all included trials. These scores will be compared against statistics reported in pre-existing meta-analyses to determine level of agreement. | We did not proceed with the RoB analysis because most trials had already been assessed in previous meta-analyses. Further, space constraints would have made it difficult to report the findings without omitting other critical analyses. We reference and comment on previously reported RoB ratings in the Conclusions. |

# SYSTEMATIC REVIEW DESCRIPTION AND SEARCH TERMS

We conducted a systematic search for medication studies published from 31 May 2015 up to 1 Jan 2021 (i.e. after the final search date of Cipriani et al.’s (2) review up to the final search date of Cuijpers et al’s (3) review). We searched PubMed, the Cochrane Central Register of Controlled Trials, Embase, Web of Science, CINAHL, PsycINFO and LiLACS for randomised controlled trials (RCTs) comparing any antidepressant with placebo in the treatment of children and adolescents with a primary diagnosis of major depressive disorder. We used the same search terms as Cipriani (2) with one additional search term to include only placebo-controlled trials (see below). We additionally applied filters to specify our date range, and to exclude reviews and non-human studies. We also searched clinical trial registers for published and unpublished studies however all RCTs meeting inclusion criteria had already been identified from the database search outlined above. Please see *Figure 1* for the PRISMA flow diagram.

We used Covidence, an online software tool, to manage our systematic review. Our search produced 538 studies, 88 of which were duplicates and subsequently removed. Two authors screened 450 titles and abstracts, and 38 full text records. Seven studies met inclusion criteria and data extraction was completed for these papers.

## Search terms

Explicit search strategy: title/abstract = (depress* or dysthymi* or “mood disorder*” or “affective disorder*“) AND (adolesc* or child* or boy* or girl* or juvenil* or minors or paediatri* or pediatri* or pubescen* or school* or student* or teen* or young or youth*) AND (selective serotonin reuptake inhibitor or SSRI or citalopram or fluoxetine or paroxetine or sertraline or escitalopram or fluvoxamine or serotonin norepinephrine reuptake inhibitor* or SNRI or venlafaxine or duloxetine or milnacipran or reboxetine or bupropion or noradrenergic and specific serotonergic antidepressants or NaSSA or mirtazapine or TCA or tricyclic or amersergide or amineptine or amitriptyline or amoxapine or butriptyline or chlorpoxiten or clomipramine or clorimipramine or demexiptiline or desipramine or dibenzipin or dothiepin or doxepin or imipramine or lofepramine or melitracen or metapramine or nortriptyline or noxiptiline or opipramol or protriptyline or quinupramine or tianeptine or trimipramine) AND (placebo)

# REFERENCES FOR INCLUDED TRIALS

References for trials included in the existing meta-analyses drawn upon for this study can be found at <https://docs.metapsy.org/databases/depression-childadol-psyctr/> for psychotherapy RCTs and at <https://ora.ox.ac.uk/objects/uuid:e0b5ae23-d562-4348-94b8-84f70b7812c5> for medication RCTs. Below are references for the seven additional RCTs identified in the original systematic review conducted for the current study.

## Publications

Atkinson, S., Lubaczewski, S., Ramaker, S., England, R. D., Wajsbrot, D. B., Abbas, R., & Findling, R. L. (2018). Desvenlafaxine versus placebo in the treatment of children and adolescents with major depressive disorder. *Journal of Child and Adolescent Psychopharmacology, 28(1),* 55-65. DOI: 10.1089/cap.2017.0099

Durgam, S., Chen, C. Z., Migliore, R., Prakash, C., Edwards, J., & Findling, R. L. (2018). A phase 3, double-blind, randomized, placebo-controlled study of vilazodone in adolescents with major depressive disorder. *Pediatric Drugs, 20(4),* 353-363. DOI: 10.1007/s40272-018-0290-4

Findling, R. L., McCusker, E., & Strawn, J. R. (2020). A randomized, double-blind, placebo-controlled trial of vilazodone in children and adolescents with major depressive disorder with twenty-six-week open-label follow-up. *Journal of Child and Adolescent Psychopharmacology, 30(6),* 355-365. DOI: 10.1089/cap.2019.0176

Le Noury, J., Nardo, J. M., Healy, D., Jureidini, J., Raven, M., Tufanaru, C., & Abi-Jaoude, E. (2015). Restoring Study 329: efficacy and harms of paroxetine and imipramine in treatment of major depression in adolescence. *BMJ, 351,*, h4320. DOI: 10.1136/bmj.h4320

Weihs, K. L., Murphy, W., Abbas, R., Chiles, D., England, R. D., Ramaker, S., & Wajsbrot, D. B. (2018). Desvenlafaxine versus placebo in a fluoxetine-referenced study of children and adolescents with major depressive disorder. Journal of Child and Adolescent Psychopharmacology, 28(1), 36-46. DOI: 10.1089/cap.2017.0100

**Unpublished clinical trials**

Active Reference (Fluoxetine) Fixed-dose Study of Vortioxetine in Paediatric Patients Aged 12 to 17 Years With Major Depressive Disorder (MDD). ClinicalTrials.gov Identifier: NCT02709746. Accessed 17 Jan 2024.

Safety and Efficacy of Levomilnacipran ER in Adolescent Participants With Major Depressive Disorder. ClinicalTrials.gov Identifier: NCT02431806. Accessed 17 Jan 2024.

# HIERARCHY OF DEPRESSION SYMPTOM SEVERITY MEASUREMENT SCALES

Where multiple depression rating scales were used, we selected the best available measure according to the following hierarchy used in Cipriani et al. (2).

1. Children’s Depression Rating Scale (CDRS)
2. Hamilton Depression Rating Scale (HAMD)
3. Montgomery Asberg Depression Rating Scale (MADRS)
4. Beck Depression Inventory (BDI)
5. Children’s Depression Inventory (CDI)
6. Schedule for Affective Disorders and Schizophrenia for School-Aged Children (K-SADS)
7. Mood and Feeling Questionnaire (MFQ)
8. Reynolds Adolescent Depression Scale (RADS)
9. Bellevue Index of Depression (BID)
10. Child Depression Scale (CDS)
11. Centre for Epidemiological Studies Depression Scale (CES-D)
12. Child Assessment Schedule (CAS)
13. Child Behaviour Checklist-Depression (CBCL-D)

##

# FULL DESCRIPTION OF METHODS (INCLUDING FORMALISMS)

## Included studies

We drew upon RCTs included in two recent comprehensive meta-analyses with open data available for each medication and psychotherapy, and supplemented them with an updated systematic review. Please refer to these original meta-analyses for a detailed description of their search strategy and study selection criteria. Psychotherapy studies were drawn from a systematic review and meta-analysis of randomised trials comparing psychotherapy for youth depression against control conditions (3) (dataset available at <https://docs.metapsy.org/databases/depression-childadol-psyctr/>). Whilst Cuijpers et al. (3) excluded studies for which the primary outcome variable could not be calculated due to missing data, we included these studies and performed the imputations outlined below. We also included studies which had data available for other variables in interest, including number of sites or baseline demographics; hence we have more psychotherapy studies included in this review compared to the original meta-analysis. Whilst the online database is regularly updated, we chose to exclude studies published after the final date of Cuijpers et al.’s (3) literature search. We also included four studies that we were able to locate via Zhou et al. (4) which were not covered in the previous psychotherapy meta-analysis (3), but which met our inclusion criteria.

Medication studies were drawn from a network meta-analysis examining the efficacy and tolerability of antidepressants and placebo for major depressive disorder in children and adolescents (2). A dataset was made available online though did not include means or standard deviations at baseline or post-test. We were unable to access the full dataset used in this meta-analysis, and hence completed extraction from the included studies ourselves. We excluded three studies because they had no control arm. We were unable to locate and therefore complete extraction for two RCTs (Almeida-Montes, 2005; Eli Lilly, 1986). Many studies did not report complete data; we contacted all corresponding authors to request missing data, though did not receive any responses.

We conducted a systematic search for medication studies published after the final search date of Cipriani et al.’s (2) review up to the final search date of Cuijpers et al’s (3) review to ensure we analysed an equivalently up-to-date database of medication trials. Please see the Supplemental Materials for further details. Our search produced 538 studies, 88 of which were duplicates and subsequently removed. Two authors screened 450 titles and abstracts, and 38 full text records. Seven studies met inclusion criteria and data extraction was completed for these papers.

## Statistical Analysis

Sample characteristics

We conducted a series of random-effects meta-analyses and tested for subgroup differences between psychotherapy and medication trials in sample characteristics including sex, age, and severity of depressive symptoms at baseline. Meta-analyses were implemented using R’s Meta package (version 7.0-0).

In order to compare depression severity across the variety of instruments the studies used, we performed a min-max normalisation to turn each study arm mean score at baseline into a percentage using the following formalism:

$$\text{outcome}_{percent}=\frac{X-\text{scale}_{min}}{\text{scale}_{max}-\text{scale}_{min}}$$

where,

$$X$$

is the mean score for each study arm on the primary outcome questionnaire, and ${scale}_{min}$ and ${scale}_{max}$ are the minimum and maximum possible values of the scale in question, respectively. The standard deviation is calculated thus:

$$\text{SD}_{Xpercent}=\frac{\text{SD}_{X}}{\text{scale}_{max}-\text{scale}_{min}}$$

where $\text{SD}_{X}$ is the original standard deviation of the mean at baseline.

Trial design

*Measures of Effect*

As the measure of effect of each individual study, we used the within-group Standardised Mean Difference (SMD), which we defined following (27) as:

$$SMD_{change}=\frac{Mean_{t_{2}}-Mean_{t_{1}}}{\frac{SD_{t2}+SD_{t1}}{2}}$$

where, $Mean_{t_{2}}$ and $Mean_{t_{1}}$ refer to the means of the main outcome score at the end and beginning of the intervention respectively and $SD_{t_{2}}$ and $SD_{t_{1}}$ to the respective standard deviations. Where individual studies did not report all data required to calculate the SMD, we imputed missing data according to the methods summarised in this Cochrane Handbook (20), in the following order. If a study reported the standard error of the mean, the SD was obtained simply by multiplying the SE by the square root of the sample size. For conditions where the SD was missing at one time point, the baseline SD was substituted by the post-test SD, and vice versa. If the SD was not available at either time point, missing values were replaced by the mean of the SDs available for comparable cases (defined as same trial type (psy or med), same instrument, same timepoint (pre or post), and same arm (control or active)). Where there were missing means at either baseline or post-test, missing values were calculated using mean change scores, preferring the change scores reported in the paper itself, though where this was unavailable, using the change scores reported in the dataset from Cipriani et al.’s meta-analysis (for medication studies only).

For the purposes of meta-analysis, it is necessary to estimate a standard error of the SMD. This is calculated according to:

$$SE_{SMD}=\sqrt{\frac{2\left( 1-r_{t_{1}t_{2}} \right)}{n}+\frac{SMD^{2}}{2n}}$$

where $n$ refers to the study sample size and $r_{t_{1}t_{2}}$ refers to the correlation between the outcome score obtained at baseline and at the end point. This correlation is typically not reported in studies and is often imputed using previously reported correlations for the instruments used. However, this practice has given rise to concerns about misestimation. Whilst such misestimation is possible, there is no reason to expect that it would be systematic, i.e. bias estimation of the effects for the control group of medication compared to those of psychotherapy. Still, to alleviate such concerns we have used simulations.

In particular, we simulated one thousand truncated distribution of standard errors with the following general characteristics:

$$r_{t_{1}t_{2}}\mathcal{\sim TN}\left( \mu,\sigma,a,b \right)$$

for which we chose the mean to be $\mu=0.65$, the standard deviation to be $sigma=0.2$, and the upper and lower bounds to be $a=0.45$ and $b=0.9$, respectively. We then used these simulated datasets in the subsequent meta-analyses.

*Multilevel model metaregression*

We estimated pooled standardized mean differences for each arm by using multilevel models implemented in R's metafor package. Unlike the traditional random effects meta-analysis, which assumes that each study's true effect size $\theta_{k}$ varies due to heterogeneity between studies, our multilevel model accounts for the hierarchical structure of the data, with study arms nested within study IDs. This allows us to model variability at both the study level and the study arm level.

The multilevel model assumes that each study's true effect size $\theta_{ij}$ (where $i$ indexes the study and $j$ indexes the study arm) is influenced by both the variability between studies and the variability between arms within each study. The model can be expressed as:

$$Y_{ij}\mathcal{\sim N}\left( \theta_{ij},\sigma_{ij}^{2} \right)$$

Where,

$$\theta_{ij}\mathcal{\sim N}\left( x_{ij}\beta,\tau_{i}^{2} \right)$$

where$Y_{ij}$ is the observed effect size for the $j$^th^ arm in the $i$^th^ study, which has a normal distribution with mean $\theta_{ij}$ and sampling error variance $\sigma_{ij}^{2}$. The true effect size $\theta_{ij}$ is modeled as a study-specific effect with an additional term representing the variability between arms within the study.

This gives rise to the following model:

$$Y_{ij}=x_{ij}\beta+u_{i}+v_{\mathrm{ij}}+\epsilon_{ij}$$

Where,

$$u_{i}\sim N\left( 0,\tau^{2} \right)$$

describes the deviation of each study from the overall mean effect size, and

$$v_{ij}\sim N\left( 0,\tau^{2} \right)$$

describes the deviation of each arm from the study-specific effect, with $\tau_{i}^{2}$ representing the heterogeneity within studies. Finally,

$$\epsilon_{ij}\sim N\left( 0,{\sigma_{ij}}^{2} \right)$$

represents the sampling error.

In this framework, we can model the means for each arm of the trials as follows:

$$\begin{matrix} \Upsilon_{ij} & =\left\{ \begin{matrix} 0 & MedControl:b_{0}+u_{i}+v_{\mathrm{ij}}+\epsilon_{ij} \\ 1 & MedActive:b_{0}+b_{1_{ij}}+u_{i}+v_{\mathrm{ij}}+\epsilon_{ij} \\ 2 & PsyActive:b_{0}+b_{2_{ij}}+u_{i}+v_{\mathrm{ij}}+\epsilon_{ij} \\ 3 & PsyControl:b_{0}+b_{3_{ij}}+u_{i}+v_{\mathrm{ij}}+\epsilon_{ij} \\ & \end{matrix} \right. \end{matrix}$$

Here, the mean effect size for each level is the sum of $b_{0}$, the intercept for the reference category (medication control), with the coefficient for each level (e.g., $b_{3_{ij}}$ for psychotherapy controls). The variability between studies and arms within studies is captured by $u_{i}$ and $v_{ij}$, respectively. The confidence intervals for the means are constructed using the standard errors of the means, which account for the hierarchical structure of the data. Each coefficient represents the contrast between the reference category and each level. For example, $b_{3_{ij}}$ represents the contrast between psychotherapy and medication control arms. Inference on these contrasts is conducted using the following test statistic:

$$z=\frac{\hat{\beta}}{\text{SE}\left( \hat{\beta} \right)}$$

This test allows us to assess the significance of the differences between treatment effects across study arms.

We used maximum likelihood (ML) to estimate model and applied Hartung-Knapp adjustment to reduce the chance of false positives (28).

We present the SMDs of each of the four treatment arms (medication control, medication active, psychotherapy control, psychotherapy active) under investigation. The SMDs are the means across the 1000 simulated datasets.

*Number of sites*

We also conducted a t-test to compare mean number of trial sites between psychotherapy and medication trials.

Sensitivity Analyses

We conducted a series of sensitivity analyses. For each of the meta-analyses we excluded studies that 1) used waitlist as their control and 2) recruited participants with subclinical levels of depression. Next, we conducted two analyses where we included only trials that used the Children’s Depression Rating Scale, Revised (CDRS-R) or the Hamilton Depression Rating Scale (HAM-D) as outcome instruments. Additionally, we restricted two analyses to studies with variance below 0.02 and which reported post SD respectively.

Further, we tested whether the simulated values for the standard error had a substantial influence on the estimation of the differences between the medication and psychotherapy control conditions. To inspect whether this is the case, we plotted the z-value of the difference between the two coefficients against the number of simulations. We make inference on the stability of the difference, by counting the proportion of times that the z-value is above the critical value of z = 1.645 corresponding to an alpha = 0.05.

Finally, we examined whether differential regression to the mean may account for differences in effect for psychotherapy and medication trials.

Comparing the control and active arms of psychotherapy trials

We ran t-tests to compare the active and control arms of psychotherapy trials on key variables of interest regarding the intensity of the interventions. We extracted data pertaining to the number, duration and intensity of sessions, and the total cumulative hours and duration of the intervention. Where a range was provided, the maximum was encoded (e.g. if a paper reported that an intervention involved 8-10 sessions lasting 50-60 minutes, we encoded the number and duration of sessions as 10 and 60, respectively). If sessions varied in frequency across an intervention, we calculated an average by dividing total number of sessions by length of intervention period. Similarly, if the length of sessions varied across the course of the intervention, we calculated a weighted average. Phone call, web-chat and online sessions were encoded as sessions, however guided self-help components were not.

# ADDITIONAL TABLES AND FIGURES

| Table S2: Summary of included RCTs   \| **Study** \| **Arm** \| **Description** \| **N** \| **Baseline M** \| **Baseline SD** \| **Post M** \| **Post SD** \| **Cohen's d** \| \| --- \| --- \| --- \| --- \| --- \| --- \| --- \| --- \| --- \| \| Atkinson, 2014 \| Active \| Duloxetine \| 117 \| 59.20 \| 10.50 \| 35.00 \| 10.50 \| -2.30 \| \| Active \| Fluoxetine \| 117 \| 58.80 \| 10.60 \| 35.60 \| 10.60 \| -2.19 \| \| Control \| Placebo \| 103 \| 60.20 \| 11.70 \| 35.00 \| 11.70 \| -2.15 \| \| Atkinson, 2018 \| Active \| Desvenlafaxine (high dose) \| 121 \| 58.45 \| 9.45 \| 34.05 \| 9.45 \| -2.58 \| \| Active \| Desvenlafaxine (low dose) \| 122 \| 58.52 \| 9.18 \| 34.82 \| 9.18 \| -2.58 \| \| Control \| Placebo \| 119 \| 57.28 \| 8.94 \| 34.38 \| 8.94 \| -2.56 \| \| Berard, 2006 \| Active \| Paroxetine \| 182 \| 25.90 \| 6.75 \| 12.30 \| 6.75 \| -2.01 \| \| Control \| Placebo \| 93 \| 25.90 \| 5.79 \| 13.10 \| 5.79 \| -2.21 \| \| Bristol-Myers Squibb, 2002a \| Active \| Nefazodone \|  \|  \| 9.33 \|  \| 10.83 \|  \| \| Control \| Placebo \|  \|  \| 9.30 \|  \| 10.87 \|  \| \| Bristol-Myers Squibb, 2002b \| Active \| Nefazodone \| 90 \| 61.20 \| 9.33 \| 38.00 \| 10.83 \| -2.30 \| \| Control \| Placebo \| 93 \| 58.30 \| 9.30 \| 36.70 \| 10.87 \| -2.14 \| \| Durgam, 2018 \| Active \| Vilazodone (15mg) \| 175 \| 57.80 \| 8.70 \| 33.80 \| 12.00 \| -2.32 \| \| Active \| Vilazodone (30mg) \| 180 \| 56.80 \| 8.50 \| 32.50 \| 11.50 \| -2.43 \| \| Control \| Placebo \| 171 \| 57.50 \| 8.60 \| 34.00 \| 12.90 \| -2.19 \| \| Emslie, 1997 \| Active \| Fluoxetine \| 48 \| 58.50 \| 10.50 \| 38.40 \| 14.80 \| -1.59 \| \| Control \| Placebo \| 48 \| 57.60 \| 10.40 \| 47.10 \| 17.00 \| -0.77 \| \| Emslie, 2002a \| Active \| Fluoxetine \| 109 \| 57.10 \| 9.90 \| 35.10 \| 13.50 \| -1.88 \| \| Control \| Placebo \| 110 \| 55.10 \| 11.80 \| 40.20 \| 13.50 \| -1.18 \| \| Emslie, 2002b \| Active \| Nefazodone \| 99 \|  \| 9.33 \|  \| 10.83 \|  \| \| Control \| Placebo \| 96 \|  \| 9.30 \|  \| 10.87 \|  \| \| Emslie, 2006 \| Active \| Paroxetine \| 101 \| 60.70 \| 9.37 \| 38.12 \| 9.37 \| -2.41 \| \| Control \| Placebo \| 102 \| 62.60 \| 8.96 \| 39.22 \| 8.96 \| -2.61 \| \| Emslie, 2007a \| Active \| Venlafaxine \| 80 \| 54.40 \| 13.19 \| 36.30 \| 13.19 \| -1.37 \| \| Control \| Placebo \| 85 \| 54.40 \| 12.82 \| 38.30 \| 12.82 \| -1.26 \| \| Emslie, 2007b \| Active \| Venlafaxine \| 102 \| 57.30 \| 13.06 \| 32.70 \| 13.06 \| -1.88 \| \| Control \| Placebo \| 94 \| 57.30 \| 13.43 \| 34.50 \| 13.43 \| -1.70 \| \| Emslie, 2009 \| Active \| Escitalopram \| 154 \| 57.60 \| 8.19 \| 35.50 \| 8.19 \| -2.70 \| \| Control \| Placebo \| 157 \| 56.00 \| 8.27 \| 37.20 \| 8.27 \| -2.27 \| \| Emslie, 2014 \| Active \| Duloxetine \| 108 \| 59.30 \| 10.90 \| 35.00 \| 10.90 \| -2.23 \| \| Active \| Fluoxetine \| 117 \| 57.90 \| 10.10 \| 36.40 \| 10.10 \| -2.13 \| \| Control \| Placebo \| 122 \| 58.20 \| 9.40 \| 37.40 \| 9.40 \| -2.21 \| \| Findling, 2009 \| Active \| Fluoxetine \| 18 \| 53.00 \| 9.84 \| 34.60 \| 13.66 \| -1.57 \| \| Control \| Placebo \| 16 \| 53.94 \| 9.84 \| 31.31 \| 13.68 \| -1.92 \| \| Findling, 2020 \| Active \| Fluoxetine \| 97 \| 58.10 \| 8.30 \| 34.70 \| 12.80 \| -2.22 \| \| Active \| Vilazodone \| 186 \| 58.50 \| 9.40 \| 37.20 \| 14.20 \| -1.81 \| \| Control \| Placebo \| 182 \| 57.70 \| 9.20 \| 37.80 \| 13.70 \| -1.74 \| \| Forest, 2020 \| Active \| Fluoxetine \| 134 \| 61.50 \| 9.33 \| 37.13 \| 10.83 \| -2.42 \| \| Active \| Levomilnacipran (40mg) \| 134 \| 61.80 \| 9.33 \| 38.52 \| 10.83 \| -2.31 \| \| Active \| Levomilnacipran (80mg) \| 138 \| 59.40 \| 9.33 \| 36.76 \| 10.83 \| -2.25 \| \| Control \| Placebo \| 140 \| 61.10 \| 9.30 \| 38.20 \| 10.87 \| -2.27 \| \| Geller, 1990 \| Active \| Nortriptyline \| 12 \| 51.30 \| 4.40 \| 34.70 \| 7.80 \| -2.72 \| \| Control \| Placebo \| 19 \| 52.40 \| 3.70 \| 37.80 \| 9.10 \| -2.28 \| \| Geller, 1992 \| Active \| Nortriptyline \| 26 \| 49.90 \| 4.20 \| 32.90 \| 11.40 \| -2.18 \| \| Control \| Placebo \| 24 \| 49.60 \| 4.60 \| 32.00 \| 9.80 \| -2.44 \| \| Hughes, 1990 \| Active \| Imipramine \|  \|  \| 9.33 \|  \| 10.83 \|  \| \| Control \| Placebo \|  \|  \| 9.30 \|  \| 10.87 \|  \| \| Keller, 2001 \| Active \| Imipramine \| 95 \| 18.11 \| 4.19 \| 9.20 \| 7.85 \| -1.48 \| \| Active \| Paroxetine \| 93 \| 18.98 \| 4.15 \| 8.24 \| 7.68 \| -1.82 \| \| Control \| Placebo \| 87 \| 18.97 \| 4.10 \| 9.88 \| 7.74 \| -1.54 \| \| Klein, 1998 \| Active \| Desipramine \| 23 \| 21.44 \| 3.70 \| 10.23 \| 2.10 \| -3.87 \| \| Control \| Placebo \| 22 \| 21.33 \| 5.20 \| 14.61 \| 2.10 \| -1.84 \| \| Kutcher, 1994 \| Active \| Desipramine \| 30 \| 22.63 \| 5.17 \| 12.68 \| 8.68 \| -1.44 \| \| Control \| Placebo \| 30 \| 23.77 \| 5.31 \| 13.42 \| 8.43 \| -1.51 \| \| Kye, 1996 \| Active \| Amitriptyline \| 18 \| 12.00 \| 4.50 \| 8.00 \| 4.90 \| -0.85 \| \| Control \| Placebo \| 13 \| 13.20 \| 4.10 \| 8.80 \| 4.50 \| -1.02 \| \| Le Noury, 2015 \| Active \| Imipramine \| 95 \| 18.10 \| 4.19 \| 9.10 \| 4.19 \| -2.15 \| \| Active \| Paroxetine \| 93 \| 18.90 \| 4.24 \| 8.20 \| 4.24 \| -2.52 \| \| Control \| Placebo \| 87 \| 19.00 \| 4.10 \| 9.90 \| 4.10 \| -2.22 \| \| Lundbeck, 2020 \| Active \| Fluoxetine \| 150 \| 61.80 \| 8.90 \| 39.80 \| 8.90 \| -2.47 \| \| Active \| Vortioxetine (10mg) \| 145 \| 61.20 \| 9.40 \| 44.10 \| 9.40 \| -1.82 \| \| Active \| Vortioxetine (20mg) \| 159 \| 62.50 \| 9.80 \| 43.60 \| 9.80 \| -1.93 \| \| Control \| Placebo \| 153 \| 60.60 \| 9.10 \| 42.40 \| 9.10 \| -2.00 \| \| March, 2004 \| Active \| Fluoxetine \| 109 \| 58.94 \| 4.00 \| 36.30 \| 8.18 \| -3.72 \| \| Control \| Placebo \| 112 \| 61.18 \| 4.27 \| 41.77 \| 7.99 \| -3.17 \| \| Organon, 2002a \| Active \| Mirtazapine \| 82 \| 50.93 \| 9.33 \| 35.08 \| 10.83 \| -1.57 \| \| Control \| Placebo \| 44 \| 51.93 \| 9.30 \| 37.24 \| 10.87 \| -1.46 \| \| Organon, 2002b \| Active \| Mirtazapine \| 88 \| 48.87 \| 9.33 \| 35.39 \| 10.83 \| -1.34 \| \| Control \| Placebo \| 45 \| 47.57 \| 9.30 \| 38.76 \| 10.87 \| -0.87 \| \| Paxil (GlaxoSmithKline), 2009 \| Active \| Paroxetine \| 29 \|  \| 9.33 \|  \| 10.83 \|  \| \| Control \| Placebo \| 27 \|  \| 9.30 \|  \| 10.87 \|  \| \| Puig-Antich, 1987 \| Active \| Imipramine \| 20 \| 3.10 \| 0.43 \| 1.90 \| 0.68 \| -2.16 \| \| Control \| Placebo \| 22 \| 3.00 \| 0.66 \| 1.90 \| 0.86 \| -1.45 \| \| Von Knorring, 2006 \| Active \| Citalopram \| 124 \| 30.00 \| 5.50 \| 17.91 \| 5.50 \| -2.20 \| \| Control \| Placebo \| 120 \| 30.00 \| 5.50 \| 18.09 \| 5.50 \| -2.17 \| \| Wagner, 2003 \| Active \| Sertraline \| 189 \| 64.30 \| 11.00 \| 41.46 \| 11.00 \| -2.08 \| \| Control \| Placebo \| 187 \| 64.60 \| 11.00 \| 44.41 \| 11.00 \| -1.84 \| \| Wagner, 2004 \| Active \| Citalopram \| 89 \| 58.80 \| 10.90 \| 37.10 \| 10.90 \| -1.99 \| \| Control \| Placebo \| 85 \| 57.80 \| 11.10 \| 41.30 \| 11.10 \| -1.49 \| \| Wagner, 2006 \| Active \| Escitalopram \| 131 \| 54.50 \| 9.33 \| 32.60 \| 10.83 \| -2.17 \| \| Control \| Placebo \| 133 \| 56.60 \| 9.30 \| 36.40 \| 10.87 \| -2.00 \| \| Weihs, 2018 \| Active \| Desvenlafaxine \| 115 \| 56.34 \| 9.59 \| 33.74 \| 9.59 \| -2.36 \| \| Active \| Fluoxetine \| 112 \| 56.26 \| 8.34 \| 31.46 \| 8.34 \| -2.97 \| \| Control \| Placebo \| 112 \| 57.06 \| 8.91 \| 33.96 \| 8.91 \| -2.59 \| \| Ackerson, 1998 \| Active \| cbt \| 12 \| 19.90 \| 5.50 \| 8.80 \| 5.30 \| -2.06 \| \| Control \| wl \| 10 \| 21.00 \| 5.00 \| 20.50 \| 3.40 \| -0.12 \| \| Arnarson, 2009 \| Active \| cbt \|  \|  \|  \|  \|  \|  \| \| Control \| cau \|  \|  \|  \|  \|  \|  \| \| Asarnow, 2002 \| Active \| cbt \|  \|  \| 7.10 \| 9.54 \| 7.10 \|  \| \| Control \| wl \|  \|  \| 10.56 \| 12.33 \| 10.56 \|  \| \| Bolton, 2007 \| Active \| ipt \| 105 \| 43.50 \| 10.10 \| 27.80 \| 17.20 \| -1.15 \| \| Control \| other ctr \| 105 \| 44.20 \| 11.20 \| 40.60 \| 15.70 \| -0.27 \| \| Control \| wl \| 104 \| 44.20 \| 10.80 \| 37.30 \| 15.90 \| -0.52 \| \| Brent, 1997 \| Active \| cbt \| 37 \| 24.30 \| 8.10 \| 5.70 \| 8.60 \| -2.23 \| \| Active \| other psy \| 35 \| 22.60 \| 8.20 \| 9.10 \| 9.10 \| -1.56 \| \| Control \| wl \| 35 \| 25.70 \| 7.80 \|  \| 7.80 \|  \| \| Charkhandeh, 2016 \| Active \| cbt \| 65 \| 29.46 \| 5.47 \| 19.94 \| 5.59 \| -1.72 \| \| Control \| other ctr \| 63 \| 30.00 \| 5.37 \| 26.33 \| 5.88 \| -0.65 \| \| Control \| wl \| 60 \| 30.35 \| 5.45 \| 30.38 \| 4.66 \| 0.01 \| \| Clarke, 1995 \| Active \| cbt \| 55 \| 3.55 \| 3.20 \| 1.87 \| 2.50 \| -0.59 \| \| Control \| cau \| 70 \| 3.86 \| 3.10 \| 2.91 \| 4.30 \| -0.26 \| \| Clarke, 1999 \| Active \| cbt \| 32 \| 15.10 \| 6.00 \| 6.70 \| 7.10 \| -1.28 \| \| Active \| cbt \| 37 \| 13.00 \| 5.30 \| 4.60 \| 4.80 \| -1.66 \| \| Control \| wl \| 27 \| 14.50 \| 5.90 \| 7.70 \| 7.00 \| -1.05 \| \| Clarke, 2001 \| Active \| cbt \| 45 \| 3.20 \| 3.40 \| 1.80 \| 2.10 \| -0.51 \| \| Control \| cau \| 49 \| 3.10 \| 3.20 \| 2.90 \| 4.60 \| -0.05 \| \| Clarke, 2002 \| Active \| cbt \| 41 \| 12.00 \| 5.30 \| 5.50 \| 5.20 \| -1.24 \| \| Control \| cau \| 47 \| 11.40 \| 5.00 \| 6.00 \| 5.10 \| -1.07 \| \| De Cuyper, 2004 \| Active \| cbt \| 9 \| 12.67 \| 6.00 \| 10.11 \| 6.03 \| -0.43 \| \| Control \| wl \| 11 \| 15.27 \| 4.54 \| 11.73 \| 5.66 \| -0.69 \| \| De Jonge-Heesen, 2020 \| Active \| cbt \| 66 \| 16.18 \| 4.92 \| 13.32 \| 7.07 \| -0.48 \| \| Control \| other ctr \| 64 \| 15.68 \| 7.08 \| 14.71 \| 9.06 \| -0.12 \| \| Diamond, 2002 \| Active \| other psy \| 16 \| 23.80 \| 7.40 \| 11.80 \| 8.80 \| -1.48 \| \| Control \| wl \| 16 \| 28.00 \| 7.10 \| 18.50 \| 11.10 \| -1.04 \| \| Diamond, 2010 \| Active \| other psy \| 35 \| 33.00 \| 9.66 \| 12.60 \| 13.88 \| -1.73 \| \| Control \| cau \| 31 \| 33.00 \| 9.37 \| 18.50 \| 15.91 \| -1.15 \| \| Esposito-Smythers, 2019 \| Active \| cbt \| 74 \| 27.00 \| 8.70 \| 13.20 \| 9.30 \| -1.53 \| \| Control \| cau \| 73 \| 26.30 \| 9.80 \| 13.80 \| 9.20 \| -1.32 \| \| Fristad, 2019 \| Active \| cbt \| 19 \| 42.00 \| 9.00 \| 30.00 \| 9.00 \| -1.33 \| \| Control \| other ctr \| 18 \| 44.00 \| 13.00 \| 31.00 \| 11.00 \| -1.08 \| \| Gillham, 2006 \| Active \| cbt \| 147 \| 13.19 \| 7.81 \| 11.55 \| 8.21 \| -0.20 \| \| Control \| cau \| 124 \| 12.57 \| 7.17 \| 11.37 \| 7.90 \| -0.16 \| \| Idsoe, 2019 \| Active \| cbt \| 133 \| 33.08 \| 9.97 \| 26.85 \| 11.82 \| -0.57 \| \| Control \| cau \| 95 \| 32.01 \| 9.75 \| 29.55 \| 10.77 \| -0.24 \| \| Israel, 2013 \| Active \| other psy \| 11 \| 20.60 \| 4.60 \| 12.50 \| 7.20 \| -1.37 \| \| Control \| cau \| 9 \| 19.70 \| 5.50 \| 19.40 \| 5.20 \| -0.06 \| \| Kahn, 1990 \| Active \| cbt \| 17 \| 31.11 \| 9.58 \| 7.29 \| 66.03 \| -0.63 \| \| Active \| other psy \| 17 \| 27.18 \| 7.84 \| 13.58 \| 7.38 \| -1.79 \| \| Control \| other ctr \| 17 \| 26.94 \| 10.83 \| 12.88 \| 10.71 \| -1.31 \| \| Control \| wl \| 17 \| 28.06 \| 9.75 \| 26.94 \| 15.41 \| -0.09 \| \| Lewinsohn, 1990 \| Active \| cbt \| 19 \| 21.26 \| 11.35 \| 6.47 \| 8.53 \| -1.49 \| \| Active \| cbt \| 21 \| 21.67 \| 11.34 \| 10.00 \| 11.91 \| -1.00 \| \| Control \| wl \| 19 \| 23.84 \| 11.43 \| 20.47 \| 10.28 \| -0.31 \| \| Liddle, 1990 \| Active \| cbt \| 11 \| 21.00 \| 4.45 \| 14.45 \| 6.74 \| -1.17 \| \| Active \| cbt \| 11 \| 21.00 \| 4.45 \| 14.45 \| 6.74 \| -1.17 \| \| Control \| other ctr \| 10 \| 22.30 \| 4.24 \| 19.30 \| 6.93 \| -0.54 \| \| Control \| wl \| 10 \| 20.70 \| 3.34 \| 16.90 \| 6.79 \| -0.75 \| \| Listug-Lunde, 2013 \| Active \| cbt \| 8 \| 21.00 \| 5.29 \| 14.38 \| 9.93 \| -0.87 \| \| Control \| cau \| 8 \| 20.37 \| 4.10 \| 13.25 \| 9.87 \| -1.02 \| \| Luby, 2012 \| Active \| other psy \| 25 \| 42.80 \| 5.80 \| 30.10 \| 11.30 \| -1.49 \| \| Control \| other ctr \| 18 \| 39.80 \| 10.30 \| 33.70 \| 10.60 \| -0.58 \| \| Makover, 2019 \| Active \| other psy \|  \|  \|  \|  \|  \|  \| \| Control \| cau \|  \|  \|  \|  \|  \|  \| \| March, 2004 \| Active \| cbt \| 111 \| 59.64 \| 4.52 \| 42.06 \| 9.18 \| -2.57 \| \| Martinovic, 2006 \| Active \| cbt \| 16 \| 5.90 \| 0.80 \| 3.30 \| 1.29 \| -2.49 \| \| Control \| cau \| 16 \| 5.70 \| 0.70 \| 5.80 \| 1.98 \| 0.07 \| \| Moeini, 2019 \| Active \| cbt \| 64 \| 24.60 \| 11.70 \| 18.50 \| 14.00 \| -0.47 \| \| Control \| cau \| 64 \| 22.30 \| 11.80 \| 21.40 \| 15.60 \| -0.07 \| \| Mufson, 1999 \| Active \| ipt \| 24 \| 19.20 \| 7.50 \| 6.30 \| 7.70 \| -1.70 \| \| Control \| other ctr \| 24 \| 18.70 \| 8.60 \| 11.80 \| 8.90 \| -0.79 \| \| Mufson, 2004 \| Active \| ipt \| 34 \| 18.90 \| 5.90 \| 8.70 \| 8.00 \| -1.47 \| \| Control \| cau \| 29 \| 18.30 \| 5.00 \| 12.80 \| 8.40 \| -0.82 \| \| Poole, 2017 \| Active \| other psy \| 27 \| 18.80 \| 1.38 \| 13.23 \| 1.47 \| -3.91 \| \| Control \| cau \| 26 \| 17.50 \| 1.35 \| 13.33 \| 1.44 \| -2.99 \| \| Reed, 1994 \| Active \| other psy \| 12 \|  \|  \|  \|  \|  \| \| Control \| other ctr \| 6 \|  \|  \|  \|  \|  \| \| Reynolds, 1986 \| Active \| cbt \| 9 \| 21.11 \| 7.75 \| 6.36 \| 3.15 \| -2.71 \| \| Control \| other ctr \| 11 \| 17.09 \| 6.36 \| 5.77 \| 4.00 \| -2.19 \| \| Control \| wl \| 10 \| 16.90 \| 5.48 \| 18.31 \| 9.82 \| 0.18 \| \| Rohde, 2004 \| Active \| cbt \| 45 \| 14.20 \| 5.20 \| 6.00 \| 6.30 \| -1.43 \| \| Control \| other ctr \| 48 \| 6.00 \| 6.30 \| 8.30 \| 5.40 \| 0.39 \| \| Rohde, 2014a \| Active \| cbt \| 126 \| 1.37 \| 0.35 \| 1.40 \| 0.32 \| 0.09 \| \| Control \| other ctr \| 124 \| 1.38 \| 0.36 \| 1.50 \| 0.41 \| 0.31 \| \| Rossello, 1999 \| Active \| cbt \| 25 \| 20.12 \| 6.95 \| 13.28 \| 7.61 \| -0.94 \| \| Active \| ipt \| 23 \| 21.21 \| 7.53 \| 10.79 \| 6.51 \| -1.48 \| \| Control \| wl \| 23 \| 20.13 \| 5.99 \| 15.83 \| 6.83 \| -0.67 \| \| Sanford, 2006 \| Active \| other psy \| 16 \|  \|  \|  \|  \|  \| \| Control \| cau \| 15 \|  \|  \|  \|  \|  \| \| Santomauro, 2016 \| Active \| cbt \| 11 \| 24.20 \| 8.97 \| 17.20 \| 8.95 \| -0.78 \| \| Control \| wl \| 12 \| 21.60 \| 11.03 \| 23.93 \| 11.58 \| 0.21 \| \| Shirk, 2014 \| Active \| cbt \| 20 \| 29.85 \| 10.56 \| 21.35 \| 11.62 \| -0.77 \| \| Control \| cau \| 23 \| 32.21 \| 12.99 \| 19.38 \| 13.47 \| -0.97 \| \| Shomaker, 2016 \| Active \| cbt \| 61 \| 25.30 \| 7.30 \| 13.40 \| 7.30 \| -1.63 \| \| Control \| other ctr \| 58 \| 24.50 \| 7.50 \| 13.80 \| 7.50 \| -1.43 \| \| Srivastava, 2020 \| Active \| cbt \| 11 \| 67.80 \| 3.10 \| 33.70 \| 11.20 \| -4.77 \| \| Control \| cau \| 10 \| 66.90 \| 3.40 \| 45.30 \| 9.70 \| -3.30 \| \| Stallard, 2012 \| Active \| cbt \| 392 \| 10.64 \| 4.91 \| 8.22 \| 6.45 \| -0.43 \| \| Control \| other ctr \| 374 \| 10.60 \| 4.67 \| 8.50 \| 5.88 \| -0.40 \| \| Control \| cau \| 298 \| 10.56 \| 4.93 \| 6.81 \| 5.70 \| -0.71 \| \| Stark, 1987 \| Active \| pst \| 10 \| 33.50 \| 10.27 \| 24.16 \| 6.01 \| -1.15 \| \| Active \| other psy \| 9 \| 37.22 \| 8.36 \| 22.91 \| 4.36 \| -2.25 \| \| Control \| wl \| 9 \| 30.33 \| 6.28 \| 28.15 \| 6.21 \| -0.35 \| \| Stice, 2008 \| Active \| cbt \| 80 \| 18.20 \| 7.53 \| 14.25 \| 8.98 \| -0.48 \| \| Active \| cbt \| 89 \| 20.03 \| 10.35 \| 10.77 \| 9.04 \| -0.96 \| \| Active \| sup \| 88 \| 20.27 \| 9.83 \| 14.67 \| 10.62 \| -0.55 \| \| Control \| cau \| 84 \| 19.60 \| 9.23 \| 16.71 \| 9.74 \| -0.30 \| \| Stikkelbroek, 2020 \| Active \| cbt \|  \|  \|  \|  \|  \|  \| \| Control \| cau \|  \|  \|  \|  \|  \|  \| \| Szigethy, 2007 \| Active \| cbt \| 22 \| 25.70 \| 10.80 \| 10.70 \| 8.00 \| -1.60 \| \| Control \| cau \| 19 \| 21.80 \| 8.10 \| 16.70 \| 11.10 \| -0.53 \| \| Tang, 2009 \| Active \| ipt \| 35 \| 32.66 \| 10.06 \| 19.97 \| 14.68 \| -1.03 \| \| Control \| cau \| 38 \| 32.32 \| 8.70 \| 31.58 \| 12.01 \| -0.07 \| \| Topooco, 2018 \| Active \| cbt \| 33 \| 33.10 \| 9.40 \| 19.90 \| 7.20 \| -1.59 \| \| Control \| other ctr \| 37 \| 32.30 \| 10.20 \| 25.20 \| 7.80 \| -0.79 \| \| Topooco, 2019 \| Active \| cbt \| 35 \| 31.60 \| 10.00 \| 16.00 \| 11.30 \| -1.46 \| \| Control \| other ctr \| 35 \| 28.80 \| 7.90 \| 24.80 \| 10.40 \| -0.44 \| \| Vostanis, 1996 \| Active \| cbt \| 29 \| 33.40 \| 12.20 \| 17.60 \| 5.20 \| -1.82 \| \| Control \| other ctr \| 28 \| 28.60 \| 14.40 \| 18.40 \| 15.80 \| -0.68 \| \| Weisz, 1997 \| Active \| cbt \| 16 \| 45.25 \| 16.01 \| 33.19 \| 10.86 \| -0.90 \| \| Control \| cau \| 32 \| 38.38 \| 11.15 \| 34.94 \| 10.93 \| -0.31 \| \| Weisz, 2009 \| Active \| cbt \| 32 \| 10.88 \| 7.91 \| 8.00 \| 6.32 \| -0.40 \| \| Control \| cau \| 25 \| 11.29 \| 7.93 \| 8.47 \| 8.44 \| -0.34 \| \| Wood, 1996 \| Active \| cbt \| 24 \| 25.80 \| 9.80 \| 14.90 \| 10.40 \| -1.08 \| \| Control \| other ctr \| 24 \| 28.70 \| 11.60 \| 19.80 \| 13.30 \| -0.71 \| \| Young, 2006 \| Active \| ipt \| 27 \| 24.10 \| 6.90 \| 6.40 \| 4.80 \| -3.03 \| \| Control \| other ctr \| 14 \| 27.40 \| 6.60 \| 17.40 \| 10.50 \| -1.17 \| \| Young, 2010 \| Active \| ipt \|  \|  \|  \|  \|  \|  \| \| Control \| cau \|  \|  \|  \|  \|  \|  \| \| Young, 2016 \| Active \| ipt \| 95 \| 15.51 \| 8.52 \| 11.12 \| 8.57 \| -0.51 \| \| Control \| other ctr \| 91 \| 15.07 \| 8.65 \| 12.62 \| 9.28 \| -0.27 \| \| Yu, 2002 \| Active \| cbt \| 104 \| 17.44 \| 9.47 \| 13.64 \| 9.01 \| -0.41 \| \| Control \| cau \| 116 \| 16.72 \| 9.29 \| 16.02 \| 10.16 \| -0.07 \| |
| --- | --- | --- | --- | --- | --- | --- | --- | --- | --- | --- | --- | --- | --- | --- | --- | --- | --- | --- | --- | --- | --- | --- | --- | --- | --- | --- | --- | --- | --- | --- | --- | --- | --- | --- | --- | --- | --- | --- | --- | --- | --- | --- | --- | --- | --- | --- | --- | --- | --- | --- | --- | --- | --- | --- | --- | --- | --- | --- | --- | --- | --- | --- | --- | --- | --- | --- | --- | --- | --- | --- | --- | --- | --- | --- | --- | --- | --- | --- | --- | --- | --- | --- | --- | --- | --- | --- | --- | --- | --- | --- | --- | --- | --- | --- | --- | --- | --- | --- | --- | --- | --- | --- | --- | --- | --- | --- | --- | --- | --- | --- | --- | --- | --- | --- | --- | --- | --- | --- | --- | --- | --- | --- | --- | --- | --- | --- | --- | --- | --- | --- | --- | --- | --- | --- | --- | --- | --- | --- | --- | --- | --- | --- | --- | --- | --- | --- | --- | --- | --- | --- | --- | --- | --- | --- | --- | --- | --- | --- | --- | --- | --- | --- | --- | --- | --- | --- | --- | --- | --- | --- | --- | --- | --- | --- | --- | --- | --- | --- | --- | --- | --- | --- | --- | --- | --- | --- | --- | --- | --- | --- | --- | --- | --- | --- | --- | --- | --- | --- | --- | --- | --- | --- | --- | --- | --- | --- | --- | --- | --- | --- | --- | --- | --- | --- | --- | --- | --- | --- | --- | --- | --- | --- | --- | --- | --- | --- | --- | --- | --- | --- | --- | --- | --- | --- | --- | --- | --- | --- | --- | --- | --- | --- | --- | --- | --- | --- | --- | --- | --- | --- | --- | --- | --- | --- | --- | --- | --- | --- | --- | --- | --- | --- | --- | --- | --- | --- | --- | --- | --- | --- | --- | --- | --- | --- | --- | --- | --- | --- | --- | --- | --- | --- | --- | --- | --- | --- | --- | --- | --- | --- | --- | --- | --- | --- | --- | --- | --- | --- | --- | --- | --- | --- | --- | --- | --- | --- | --- | --- | --- | --- | --- | --- | --- | --- | --- | --- | --- | --- | --- | --- | --- | --- | --- | --- | --- | --- | --- | --- | --- | --- | --- | --- | --- | --- | --- | --- | --- | --- | --- | --- | --- | --- | --- | --- | --- | --- | --- | --- | --- | --- | --- | --- | --- | --- | --- | --- | --- | --- | --- | --- | --- | --- | --- | --- | --- | --- | --- | --- | --- | --- | --- | --- | --- | --- | --- | --- | --- | --- | --- | --- | --- | --- | --- | --- | --- | --- | --- | --- | --- | --- | --- | --- | --- | --- | --- | --- | --- | --- | --- | --- | --- | --- | --- | --- | --- | --- | --- | --- | --- | --- | --- | --- | --- | --- | --- | --- | --- | --- | --- | --- | --- | --- | --- | --- | --- | --- | --- | --- | --- | --- | --- | --- | --- | --- | --- | --- | --- | --- | --- | --- | --- | --- | --- | --- | --- | --- | --- | --- | --- | --- | --- | --- | --- | --- | --- | --- | --- | --- | --- | --- | --- | --- | --- | --- | --- | --- | --- | --- | --- | --- | --- | --- | --- | --- | --- | --- | --- | --- | --- | --- | --- | --- | --- | --- | --- | --- | --- | --- | --- | --- | --- | --- | --- | --- | --- | --- | --- | --- | --- | --- | --- | --- | --- | --- | --- | --- | --- | --- | --- | --- | --- | --- | --- | --- | --- | --- | --- | --- | --- | --- | --- | --- | --- | --- | --- | --- | --- | --- | --- | --- | --- | --- | --- | --- | --- | --- | --- | --- | --- | --- | --- | --- | --- | --- | --- | --- | --- | --- | --- | --- | --- | --- | --- | --- | --- | --- | --- | --- | --- | --- | --- | --- | --- | --- | --- | --- | --- | --- | --- | --- | --- | --- | --- | --- | --- | --- | --- | --- | --- | --- | --- | --- | --- | --- | --- | --- | --- | --- | --- | --- | --- | --- | --- | --- | --- | --- | --- | --- | --- | --- | --- | --- | --- | --- | --- | --- | --- | --- | --- | --- | --- | --- | --- | --- | --- | --- | --- | --- | --- | --- | --- | --- | --- | --- | --- | --- | --- | --- | --- | --- | --- | --- | --- | --- | --- | --- | --- | --- | --- | --- | --- | --- | --- | --- | --- | --- | --- | --- | --- | --- | --- | --- | --- | --- | --- | --- | --- | --- | --- | --- | --- | --- | --- | --- | --- | --- | --- | --- | --- | --- | --- | --- | --- | --- | --- | --- | --- | --- | --- | --- | --- | --- | --- | --- | --- | --- | --- | --- | --- | --- | --- | --- | --- | --- | --- | --- | --- | --- | --- | --- | --- | --- | --- | --- | --- | --- | --- | --- | --- | --- | --- | --- | --- | --- | --- | --- | --- | --- | --- | --- | --- | --- | --- | --- | --- | --- | --- | --- | --- | --- | --- | --- | --- | --- | --- | --- | --- | --- | --- | --- | --- | --- | --- | --- | --- | --- | --- | --- | --- | --- | --- | --- | --- | --- | --- | --- | --- | --- | --- | --- | --- | --- | --- | --- | --- | --- | --- | --- | --- | --- | --- | --- | --- | --- | --- | --- | --- | --- | --- | --- | --- | --- | --- | --- | --- | --- | --- | --- | --- | --- | --- | --- | --- | --- | --- | --- | --- | --- | --- | --- | --- | --- | --- | --- | --- | --- | --- | --- | --- | --- | --- | --- | --- | --- | --- | --- | --- | --- | --- | --- | --- | --- | --- | --- | --- | --- | --- | --- | --- | --- | --- | --- | --- | --- | --- | --- | --- | --- | --- | --- | --- | --- | --- | --- | --- | --- | --- | --- | --- | --- | --- | --- | --- | --- | --- | --- | --- | --- | --- | --- | --- | --- | --- | --- | --- | --- | --- | --- | --- | --- | --- | --- | --- | --- | --- | --- | --- | --- | --- | --- | --- | --- | --- | --- | --- | --- | --- | --- | --- | --- | --- | --- | --- | --- | --- | --- | --- | --- | --- | --- | --- | --- | --- | --- | --- | --- | --- | --- | --- | --- | --- | --- | --- | --- | --- | --- | --- | --- | --- | --- | --- | --- | --- | --- | --- | --- | --- | --- | --- | --- | --- | --- | --- | --- | --- | --- | --- | --- | --- | --- | --- | --- | --- | --- | --- | --- | --- | --- | --- | --- | --- | --- | --- | --- | --- | --- | --- | --- | --- | --- | --- | --- | --- | --- | --- | --- | --- | --- | --- | --- | --- | --- | --- | --- | --- | --- | --- | --- | --- | --- | --- | --- | --- | --- | --- | --- | --- | --- | --- | --- | --- | --- | --- | --- | --- | --- | --- | --- | --- | --- | --- | --- | --- | --- | --- | --- | --- | --- | --- | --- | --- | --- | --- | --- | --- | --- | --- | --- | --- | --- | --- | --- | --- | --- | --- | --- | --- | --- | --- | --- | --- | --- | --- | --- | --- | --- | --- | --- | --- | --- | --- | --- | --- | --- | --- | --- | --- | --- | --- | --- | --- | --- | --- | --- | --- | --- | --- | --- | --- | --- | --- | --- | --- | --- | --- | --- | --- | --- | --- | --- | --- | --- | --- | --- | --- | --- | --- | --- | --- | --- | --- | --- | --- | --- | --- | --- | --- | --- | --- | --- | --- | --- | --- | --- | --- | --- | --- | --- | --- | --- | --- | --- | --- | --- | --- | --- | --- | --- | --- | --- | --- | --- | --- | --- | --- | --- | --- | --- | --- | --- | --- | --- | --- | --- | --- | --- | --- | --- | --- | --- | --- | --- | --- | --- | --- | --- | --- | --- | --- | --- | --- | --- | --- | --- | --- | --- | --- | --- | --- | --- | --- | --- | --- | --- | --- | --- | --- | --- | --- | --- | --- | --- | --- | --- | --- | --- | --- | --- | --- | --- | --- | --- | --- | --- | --- | --- | --- | --- | --- | --- | --- | --- | --- | --- | --- | --- | --- | --- | --- | --- | --- | --- | --- | --- | --- | --- | --- | --- | --- | --- | --- | --- | --- | --- | --- | --- | --- | --- | --- | --- | --- | --- | --- | --- | --- | --- | --- | --- | --- | --- | --- | --- | --- | --- | --- | --- | --- | --- | --- | --- | --- | --- | --- | --- | --- | --- | --- | --- | --- | --- | --- | --- | --- | --- | --- | --- | --- | --- | --- | --- | --- | --- | --- | --- | --- | --- | --- | --- | --- | --- | --- | --- | --- | --- | --- | --- | --- | --- | --- | --- | --- | --- | --- | --- | --- | --- | --- | --- | --- | --- | --- | --- | --- | --- | --- | --- | --- | --- | --- | --- | --- | --- | --- | --- | --- | --- | --- | --- | --- | --- | --- | --- | --- | --- | --- | --- | --- | --- | --- | --- | --- | --- | --- | --- | --- | --- | --- | --- | --- | --- | --- | --- | --- | --- | --- | --- | --- | --- | --- | --- | --- | --- | --- | --- | --- | --- | --- | --- | --- | --- | --- | --- | --- | --- | --- | --- | --- | --- | --- | --- | --- | --- | --- | --- | --- | --- | --- | --- | --- | --- | --- | --- | --- | --- | --- | --- | --- | --- | --- | --- | --- | --- | --- | --- | --- | --- | --- | --- | --- | --- | --- | --- | --- | --- | --- | --- | --- | --- | --- | --- | --- | --- | --- | --- | --- | --- | --- | --- | --- | --- | --- | --- | --- | --- | --- | --- | --- | --- | --- | --- | --- | --- | --- | --- | --- | --- | --- | --- | --- | --- | --- | --- | --- | --- | --- | --- | --- | --- | --- | --- | --- | --- | --- | --- | --- | --- | --- | --- | --- | --- | --- | --- | --- | --- | --- | --- | --- | --- | --- | --- | --- | --- | --- | --- | --- | --- | --- | --- | --- | --- | --- | --- | --- | --- | --- | --- | --- | --- | --- | --- | --- | --- | --- | --- | --- | --- | --- | --- | --- | --- | --- | --- | --- | --- | --- | --- | --- | --- | --- | --- | --- | --- | --- | --- | --- | --- | --- | --- | --- | --- | --- | --- | --- | --- | --- | --- | --- | --- | --- | --- | --- | --- | --- | --- | --- | --- | --- | --- | --- | --- | --- | --- | --- | --- | --- | --- | --- | --- | --- | --- | --- | --- | --- | --- | --- | --- | --- | --- | --- | --- | --- | --- | --- | --- | --- | --- | --- | --- | --- | --- | --- | --- | --- | --- | --- | --- | --- | --- | --- | --- | --- | --- | --- | --- | --- | --- | --- | --- | --- | --- | --- | --- | --- | --- | --- | --- | --- | --- | --- | --- | --- | --- | --- | --- | --- | --- | --- | --- | --- | --- | --- | --- | --- | --- | --- | --- | --- | --- | --- | --- | --- | --- | --- | --- | --- | --- | --- | --- | --- | --- | --- | --- | --- | --- | --- | --- | --- | --- | --- | --- | --- | --- | --- | --- | --- | --- | --- | --- | --- | --- | --- | --- | --- | --- | --- | --- | --- | --- | --- | --- | --- | --- | --- | --- | --- | --- | --- | --- | --- | --- | --- | --- | --- | --- | --- | --- | --- | --- | --- | --- | --- | --- | --- | --- | --- | --- | --- | --- | --- | --- | --- | --- | --- | --- | --- | --- | --- | --- | --- | --- | --- | --- | --- | --- | --- | --- | --- | --- | --- | --- | --- | --- | --- | --- | --- | --- | --- | --- | --- | --- | --- | --- | --- | --- | --- | --- | --- | --- | --- | --- | --- | --- | --- | --- | --- | --- | --- | --- | --- | --- | --- | --- | --- | --- | --- | --- | --- | --- | --- | --- | --- | --- | --- | --- | --- | --- | --- | --- | --- | --- | --- | --- | --- | --- | --- | --- | --- | --- | --- | --- | --- | --- | --- | --- | --- | --- | --- | --- | --- | --- | --- | --- | --- | --- | --- | --- | --- | --- | --- | --- | --- | --- | --- | --- | --- | --- | --- | --- | --- | --- | --- | --- | --- | --- | --- | --- | --- | --- | --- | --- | --- | --- | --- | --- | --- | --- | --- | --- |

##

| Table S3: HAM-D scores at baseline across psychotherapy and medication RCTs   \| **Subgroup** \| **K** \| **Mean** \| **SE** \| **Lower CI** \| **Upper CI** \| **T2** \| **p-value** \| \| --- \| --- \| --- \| --- \| --- \| --- \| --- \| --- \| \|  \|  \|  \|  \|  \|  \|  \| 0.025 \| \| Psychotherapy \| 10 \| 12.6 \| 2.16 \| 7.72 \| 17.48 \| 46.04 \|  \| \| Medication \| 5 \| 18.89 \| 1.8 \| 13.9 \| 23.89 \| 15.65 \|  \| |
| --- | --- | --- | --- | --- | --- | --- | --- | --- | --- | --- | --- | --- | --- | --- | --- | --- | --- | --- | --- | --- | --- | --- | --- | --- | --- | --- | --- | --- | --- | --- | --- | --- |
| Table S4: CDRS-R scores at baseline across psychotherapy and medication RCTs   \| **Subgroup** \| **K** \| **Mean** \| **SE** \| **Lower CI** \| **Upper CI** \| **T2** \| **p-value** \| \| --- \| --- \| --- \| --- \| --- \| --- \| --- \| --- \| \|  \|  \|  \|  \|  \|  \|  \| 0.15 \| \| Psychotherapy \| 4 \| 46.23 \| 7.36 \| 22.81 \| 69.65 \| 215.43 \|  \| \| Medication \| 23 \| 56.89 \| 0.82 \| 55.19 \| 58.59 \| 14.94 \|  \| |

Table S5: Percentage female at baseline across psychotherapy and medication RCTs: including studies with female-only samples

| **Subgroup** | **K** | **Mean** | **SE** | **Lower CI** | **Upper CI** | **T^2^** | **p-value** |
| --- | --- | --- | --- | --- | --- | --- | --- |
| **Overall** |  |  |  |  |  |  | **0.007** |
| Psychotherapy | 51 | 62.87 | 2.46 | 57.94 | 67.81 | 307.93 |  |
| Medication | 28 | 53.72 | 2.33 | 48.94 | 58.51 | 152.15 |  |
| **Excluding subclinical** |  |  |  |  |  |  | **0.013** |
| Psychotherapy | 43 | 62.61 | 2.71 | 57.13 | 68.09 | 316.95 |  |
| Medication | 28 | 53.72 | 2.33 | 48.94 | 58.51 | 152.15 |  |
| **Excluding waitlist** |  |  |  |  |  |  | **0.009** |
| Psychotherapy | 43 | 63.17 | 2.78 | 57.56 | 68.78 | 332.09 |  |
| Medication | 28 | 53.72 | 2.33 | 48.94 | 58.51 | 152.15 |  |

## Multi-level metaregression results

In *Table S6*, we present the regression that tests our hypothesis about differences between medication and psychotherapy controls. Here, medication control is the reference category to which all others are compared. The strongest difference between arms, as judged by the z-value, is between the psychotherapy and medication controls with a z-value of 9.34 (p<0.0001).

| Table S6: Results from metaregression with overall sample   \| **Condition** \| **Coefficient** \| **SE** \| **z value** \| **Lower CI** \| **Upper CI** \| \| --- \| --- \| --- \| --- \| --- \| --- \| \| Medication Control \| -1.89 \| 0.11 \|  \| -2.10 \| -1.67 \| \| Medication Active \| -0.19 \| 0.08 \| -2.33 \| -0.35 \| -0.03 \| \| Psychotherapy Control \| 1.30 \| 0.14 \| 9.34 \| 1.02 \| 1.57 \| \| Psychotherapy Active \| 0.71 \| 0.14 \| 5.14 \| 0.44 \| 0.98 \| \| *T^2^*_Level 3_  = 0.2; *T*^2^_Level 2_ = 0.1; *I^2^_Level 3_* = 72.8; *I^2^_Level 2_* = 20.9; *K* = 189. \| \| \| \| \| \| |
| --- | --- | --- | --- | --- | --- | --- | --- | --- | --- | --- | --- | --- | --- | --- | --- | --- | --- | --- | --- | --- | --- | --- | --- | --- | --- | --- | --- | --- | --- | --- | --- | --- | --- | --- | --- | --- |

*Figure S1: Post-pre differences in mean min-max outcome scores in control arms*


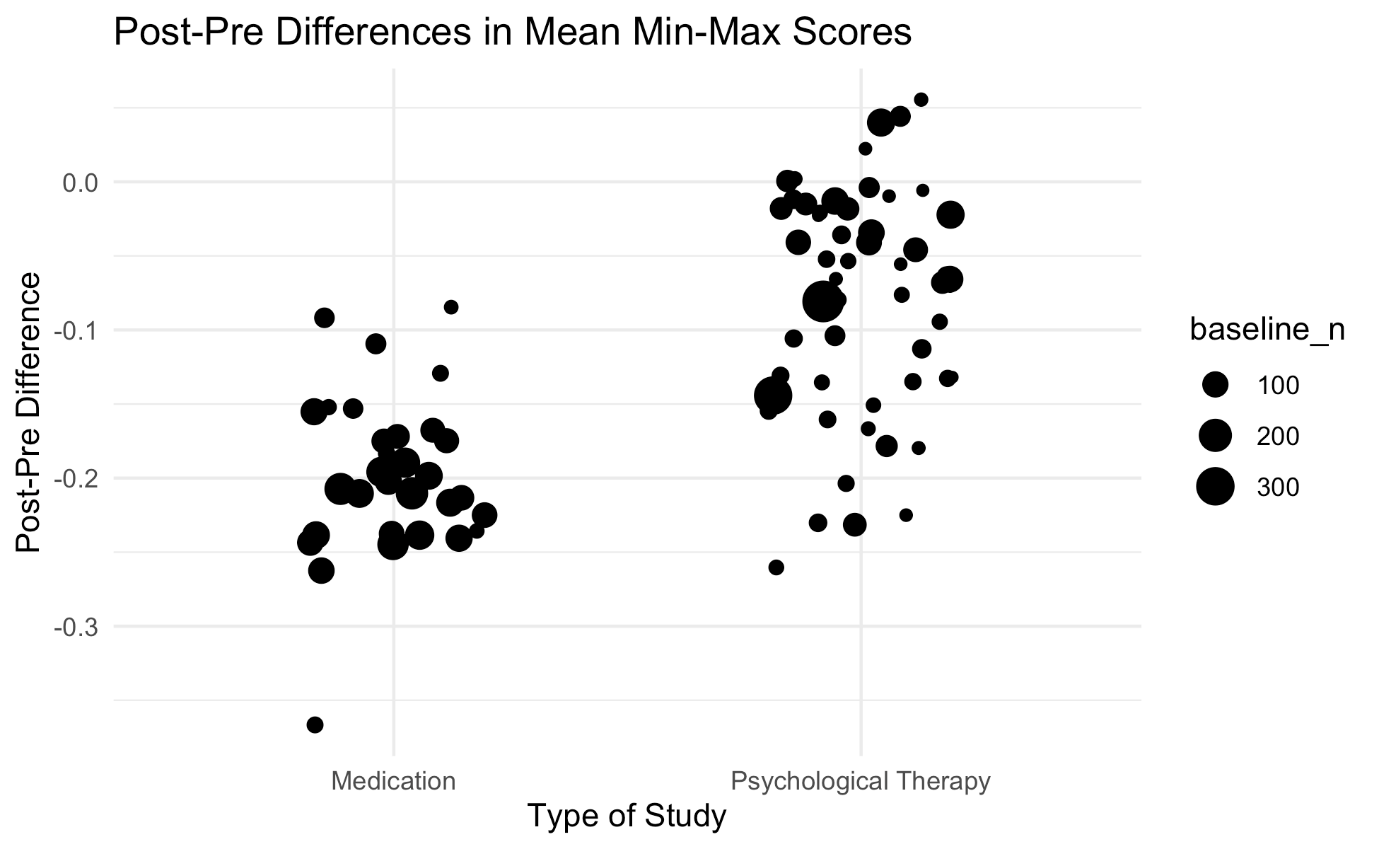


## *Figure S2: Post-pre differences in study baseline variances in control arms*


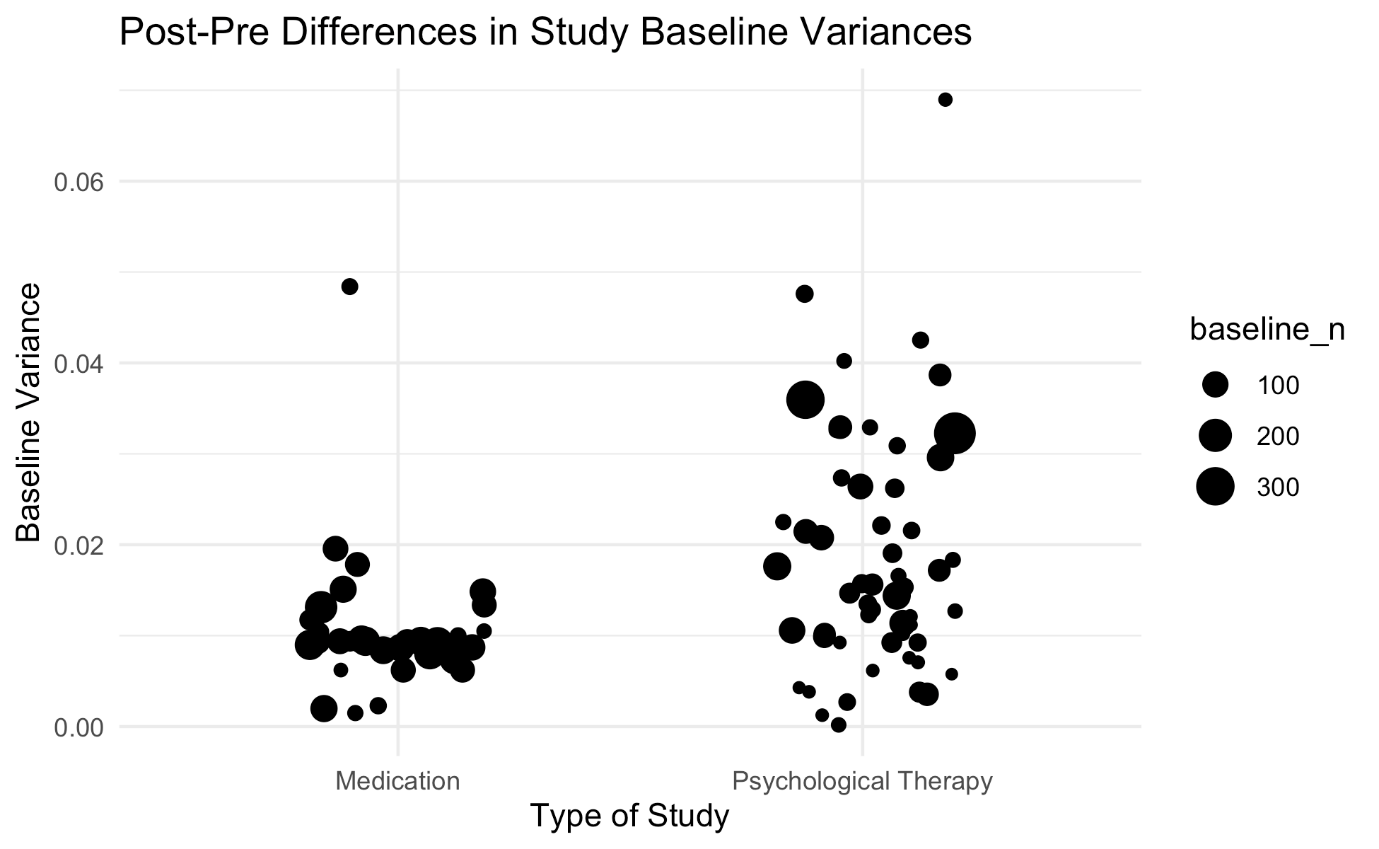


## Sensitivity analyses

| Figure S3: Meta-analytic estimates of within-group changes: waitlist studies excluded  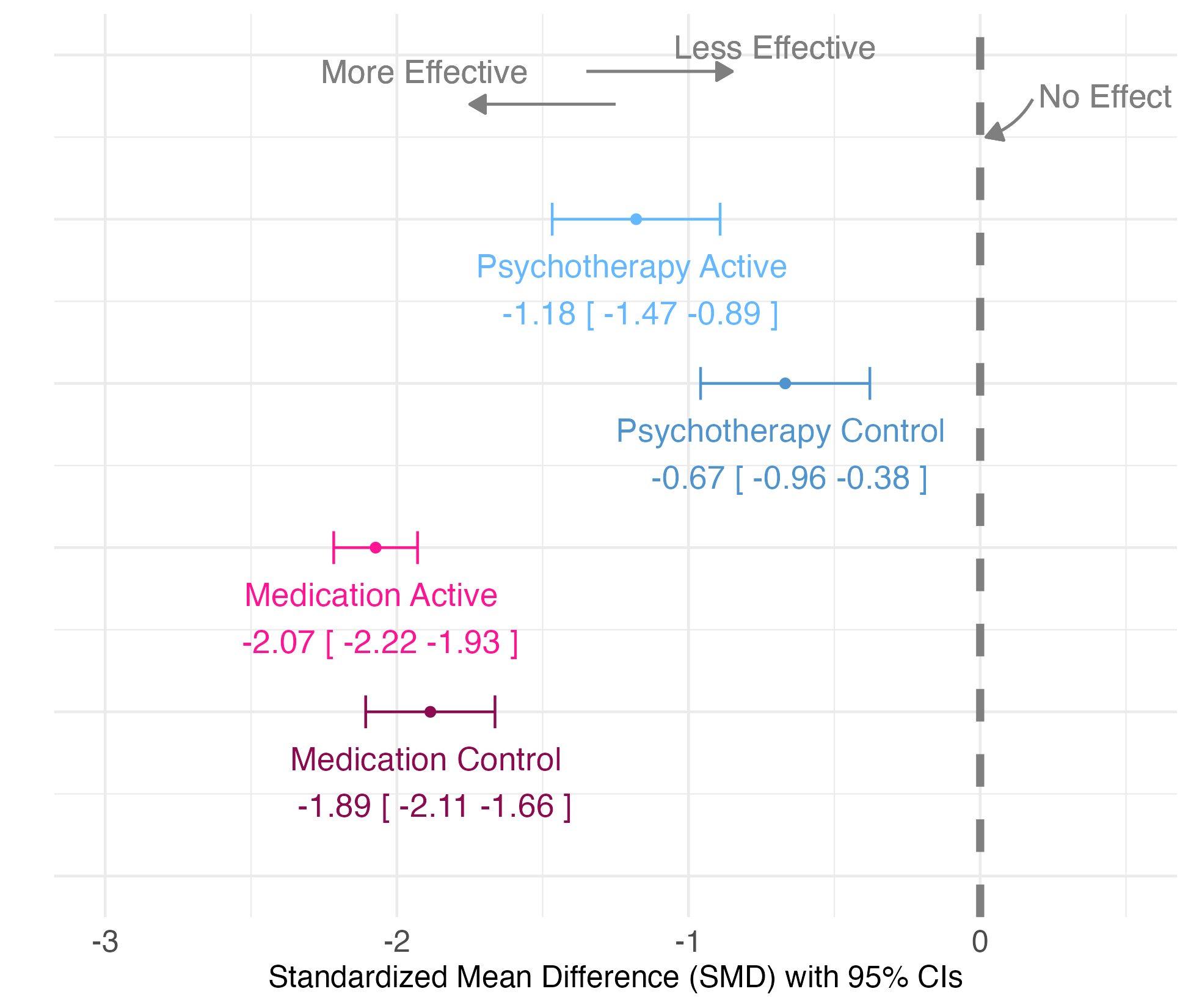 |
| --- |

| Figure S4: Meta-analytic estimates of within-group changes: subclinical studies excluded  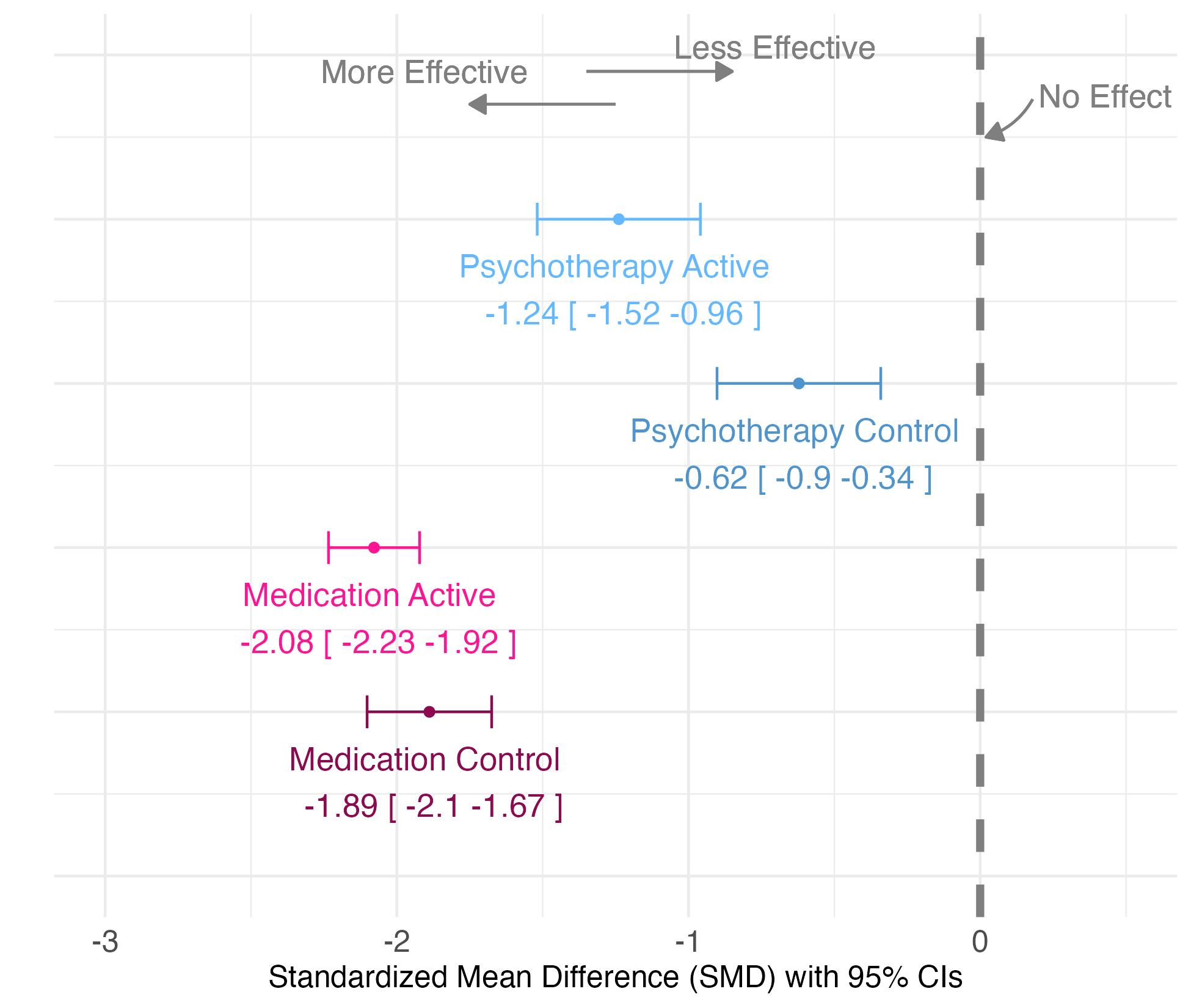 |
| --- |

| Figure S5: Meta-analytic estimates of within-group changes: CDRS studies only  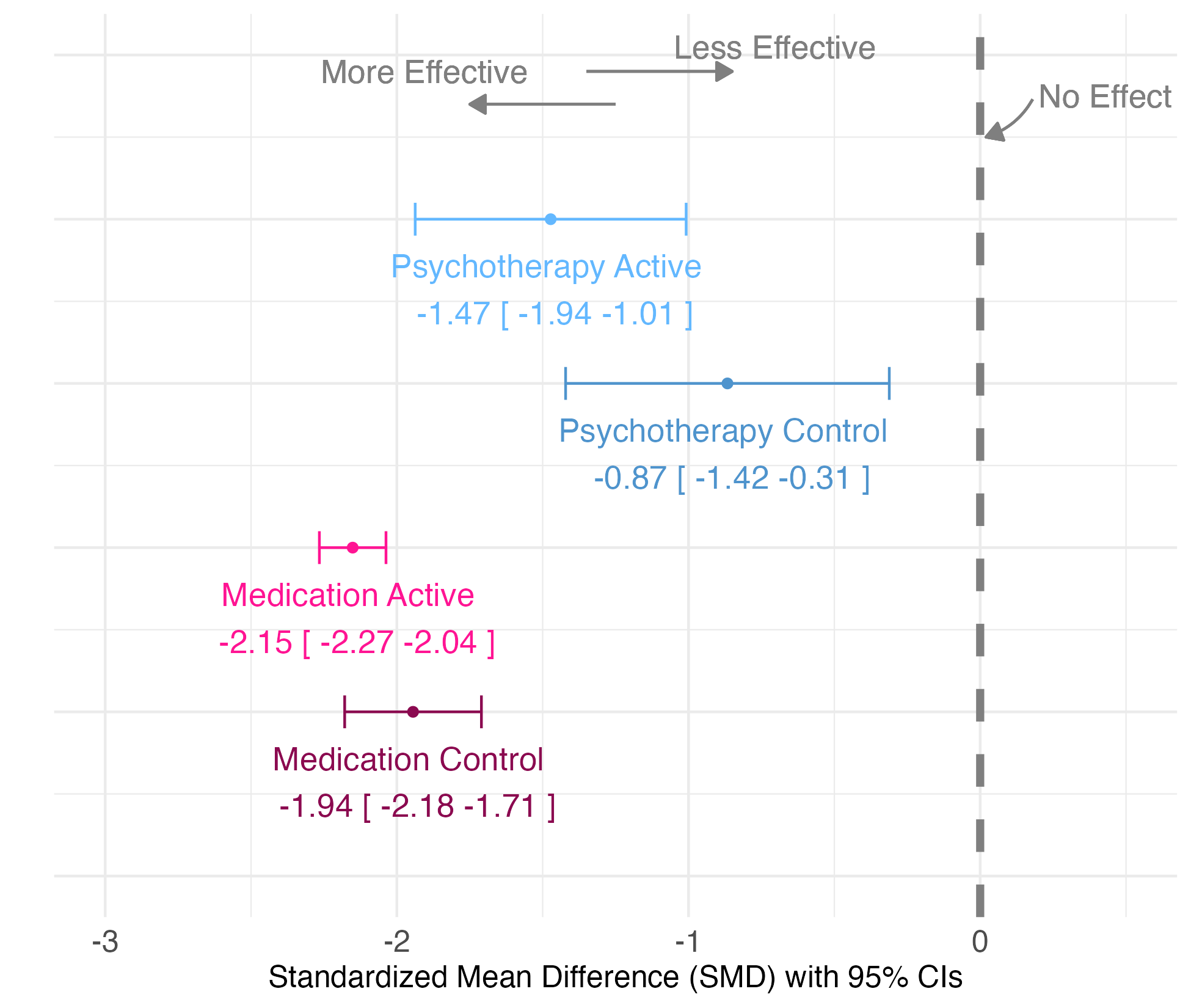 |
| --- |

| Figure S6: Meta-analytic estimates of within-group changes: HAM-D studies only  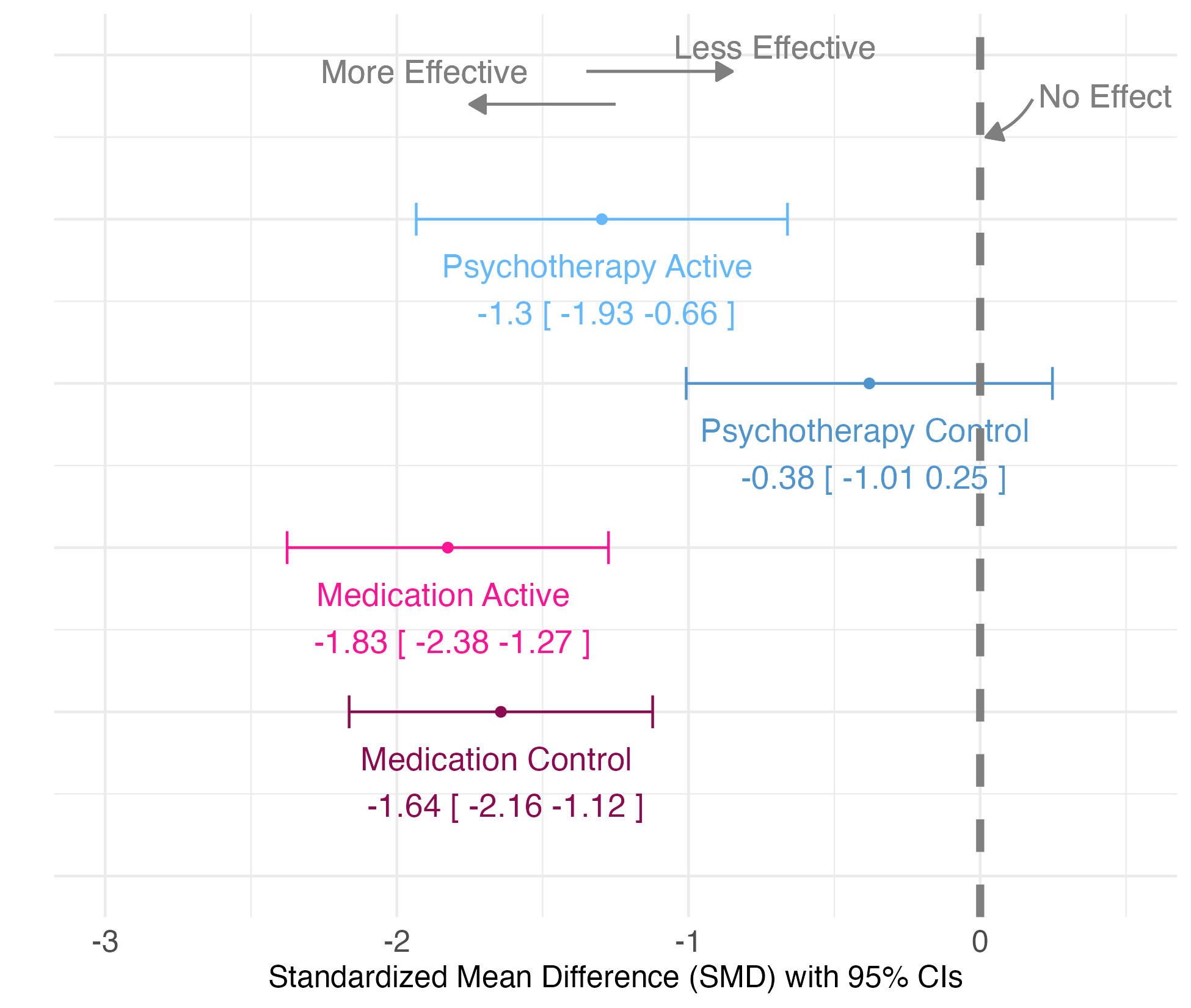 |
| --- |

####

Figure S7: Meta-analytic estimates of within-group changes: studies reporting post SD


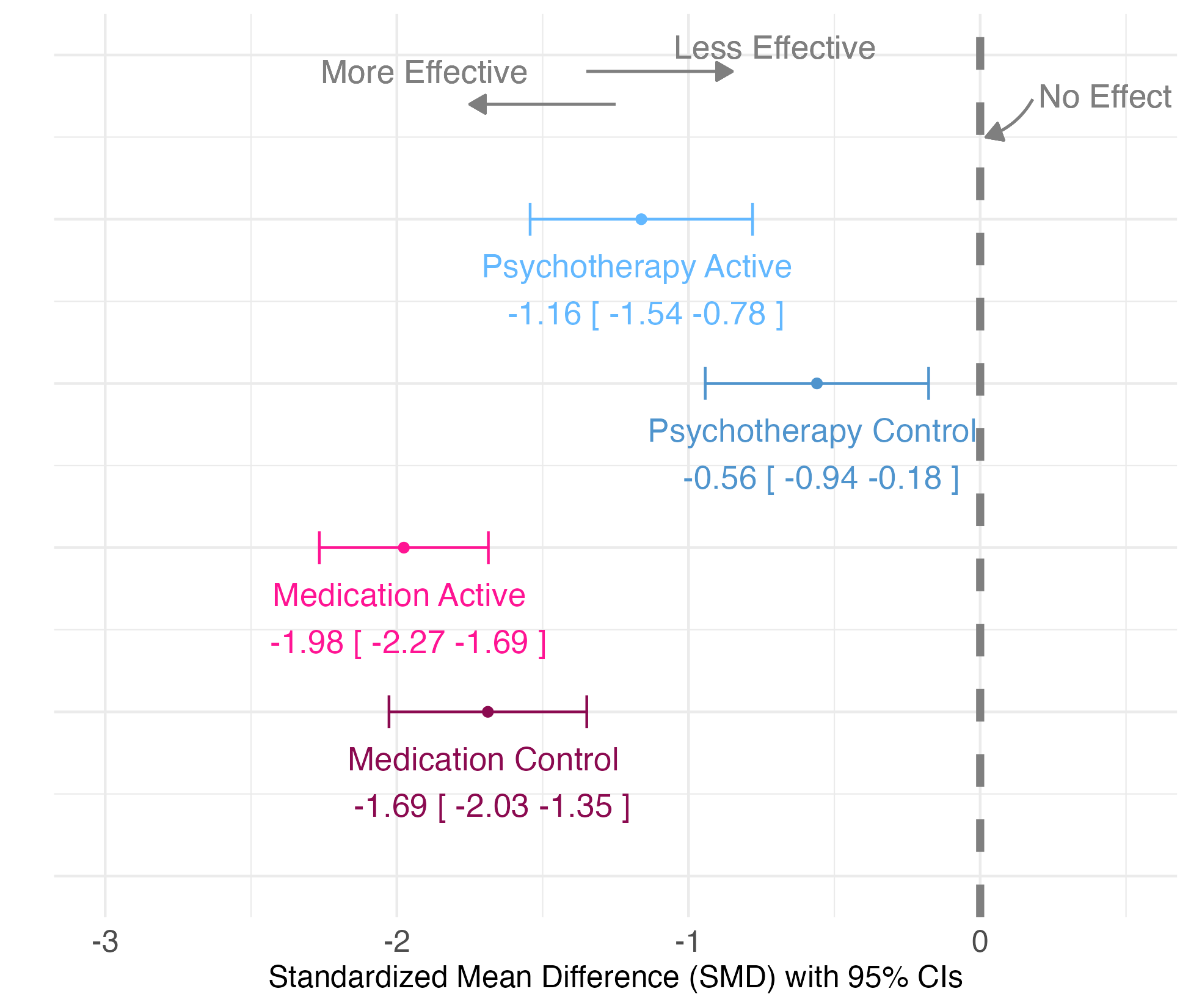


Figure S8: Meta-analytic estimates of within-group changes: studies with variance below 0.02


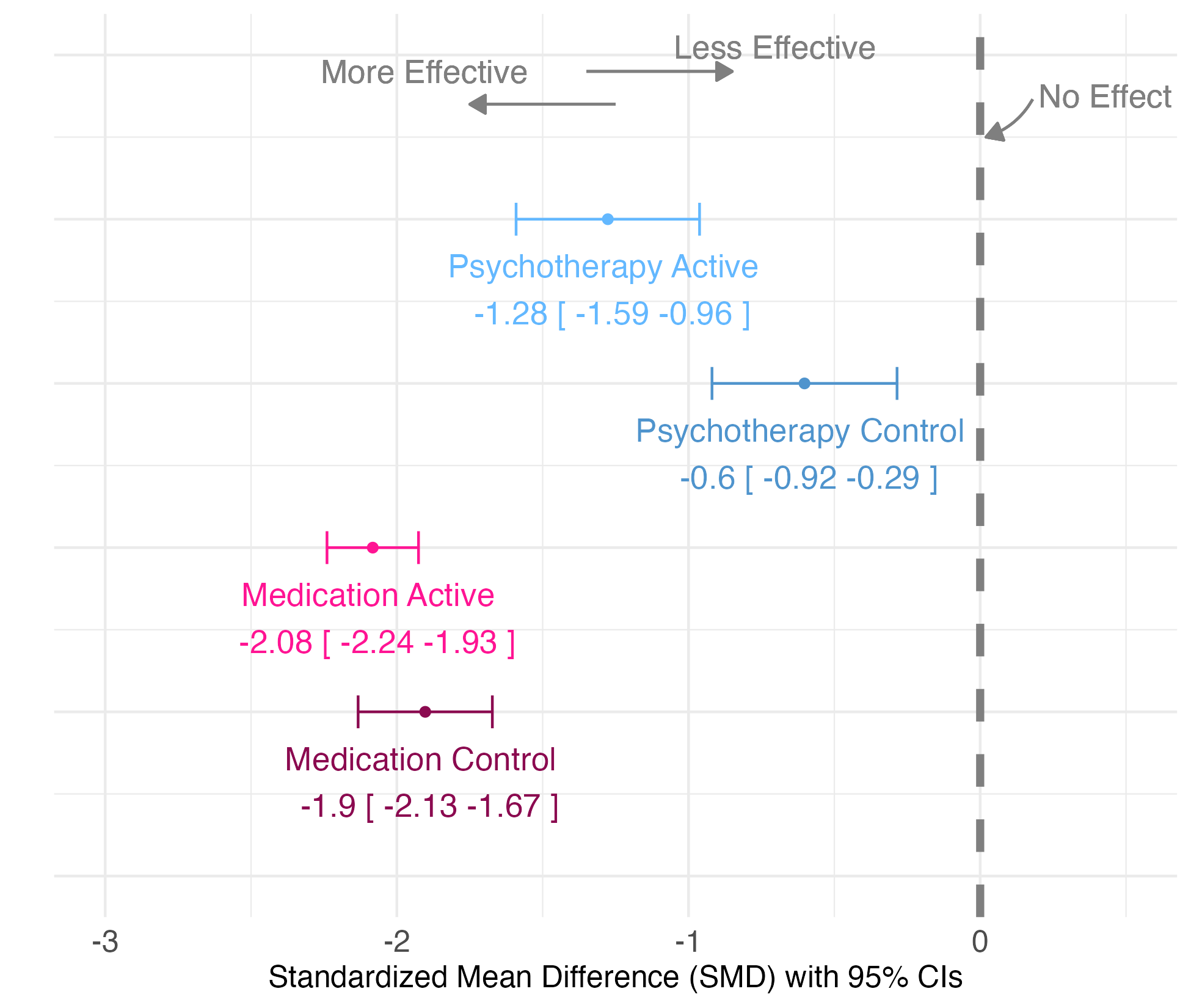


## Effect of standard errors of the SMDs

It could be argued that the choice of standard errors of the changes for the calculation of the confidence intervals could have affect the results in one or the other direction. To address such concerns we have simulated 1000 different datasets with SMDs coming from a broad distribution. If standard error distributions were influential, this should show up as substantial variability across simulations. We test this idea in the [Figure](#fig-stab-sims) S9 which displays across the 1000 simulations the z-value of the contrast between medication and psychotherapy control arms (the mean of which we presented in *Table S3*). As can be seen, the variability in the z-score is minimal and consistently far away from the threshold for significance, i.e. the value of z = 1.645.

| Figure S9: Stability of the Statistic of the Difference between Medication and Psychotherapy Control Arms  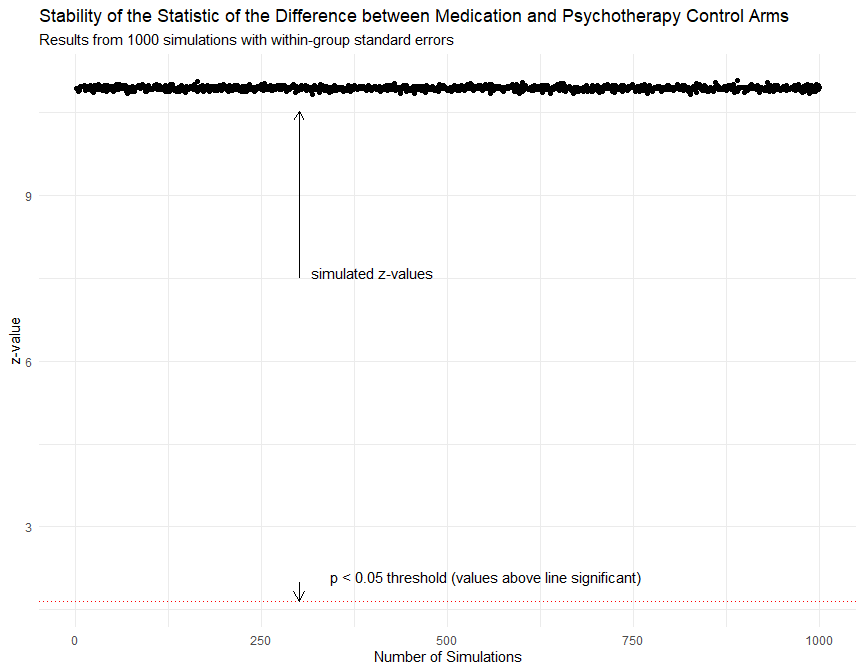 |
| --- |
|  |
|  |

| Baseline-adjusted model Table S7: Standardised mean differences (SMD) from baseline-adjusted regression model   \| **Condition** \| **SMDs** \| **Lower CI** \| **Upper CI** \| \| --- \| --- \| --- \| --- \| \| Medication Control \| 0.20 \| 0.18 \| 0.22 \| \| Medication Active \| 0.18 \| 0.16 \| 0.21 \| \| Psychotherapy Control \| 0.30 \| 0.28 \| 0.33 \| \| Psychotherapy Active \| 0.22 \| 0.20 \| 0.24 \| |
| --- | --- | --- | --- | --- | --- | --- | --- | --- | --- | --- | --- | --- | --- | --- | --- | --- | --- | --- | --- | --- |

Table S8: Estimates from baseline-adjusted regression model

| **Condition** | **Estimate** | **SE** | **t value** | **p-value** |
| --- | --- | --- | --- | --- |
| Medication Control | 0.20 | 0.01 | 20.06 | < 0.001 |
| Medication Active | -0.02 | 0.01 | -1.68 | 0.095 |
| Psychotherapy Control | 0.10 | 0.01 | 7.70 | < 0.001 |
| Psychotherapy Active | 0.02 | 0.01 | 1.27 | 0.205 |

| Table S9: Comparing the intensity of active and control arms in psychotherapy studies: waitlist studies excluded   \| **Group** \| **N** \| **Mean** \| **SD** \| **Cohen's d** \| **Upper CI** \| **Lower CI** \| **t** \| **df** \| **p-value** \| \| --- \| --- \| --- \| --- \| --- \| --- \| --- \| --- \| --- \| --- \| \| **Number of sessions** \| \| \| \| \| \| \| \| \| \| \| Active \| 52 \| 13.17 \| 12.26 \| 0.40 \| -0.09 \| 0.89 \| 2.07 \| 74.97 \| 0.042 \| \| Control \| 25 \| 8.93 \| 5.70 \|  \|  \|  \|  \|  \|  \| \| **Intensity (sessions per week)** \| \| \| \| \| \| \| \| \| \| \| Active \| 46 \| 1.20 \| 0.63 \| 0.50 \| -0.04 \| 1.03 \| 1.83 \| 36.09 \| 0.076 \| \| Control \| 21 \| 0.88 \| 0.68 \|  \|  \|  \|  \|  \|  \| \| **Session length (mins)** \| \| \| \| \| \| \| \| \| \| \| Active \| 42 \| 62.49 \| 30.20 \| 0.56 \| 0.02 \| 1.10 \| 2.02 \| 36.41 \| 0.051 \| \| Control \| 21 \| 44.93 \| 33.71 \|  \|  \|  \|  \|  \|  \| \| **Total intervention hours** \| \| \| \| \| \| \| \| \| \| \| Active \| 44 \| 12.32 \| 8.02 \| 0.46 \| -0.07 \| 0.99 \| 1.74 \| 40.57 \| 0.089 \| \| Control \| 22 \| 8.56 \| 8.36 \|  \|  \|  \|  \|  \|  \| |
| --- | --- | --- | --- | --- | --- | --- | --- | --- | --- | --- | --- | --- | --- | --- | --- | --- | --- | --- | --- | --- | --- | --- | --- | --- | --- | --- | --- | --- | --- | --- | --- | --- | --- | --- | --- | --- | --- | --- | --- | --- | --- | --- | --- | --- | --- | --- | --- | --- | --- | --- | --- | --- | --- | --- | --- | --- | --- | --- | --- | --- | --- | --- | --- | --- | --- | --- | --- | --- | --- | --- | --- | --- | --- | --- | --- | --- | --- | --- | --- | --- | --- | --- | --- | --- | --- | --- | --- | --- | --- | --- | --- | --- | --- | --- | --- | --- | --- | --- | --- | --- | --- | --- | --- | --- | --- | --- | --- | --- | --- | --- | --- | --- | --- | --- | --- | --- | --- | --- | --- | --- | --- | --- | --- | --- | --- | --- | --- | --- | --- | --- |
